# Flight Performance and Flight in Wind in Three Phylogenetically Distinct Miniature Insects

**DOI:** 10.1093/icb/icag099

**Published:** 2026-06-22

**Authors:** Amir Sarig, Lior Gurka, Gal Ribak

**Affiliations:** School of Zoology, Faculty of Life Sciences, Tel Aviv University 6997801, Israel; School of Zoology, Faculty of Life Sciences, Tel Aviv University 6997801, Israel; School of Zoology, Faculty of Life Sciences, Tel Aviv University 6997801, Israel

## Abstract

Small size imposes constraints on flight due to a reduction in aerodynamic efficiency of small wings. Consequently, miniature insects are often considered to be poor flyers that disperse by drifting in winds. Only few studies have actually measured the active flight of very small insects. The few that did, report surprisingly high flight speeds compared to the size of such small creatures. To test both the limits to flight performance and response to wind, we tracked free-flight within a wind tunnel in a whitefly, a thrips, and a bark beetle. The studied species represent small insects from three different orders (Hemiptera, Thysanoptera and Coleoptera) that underwent independent miniaturization in the course of evolution. Flights were tracked with and without wind and the wind was either horizontal or vertical. From the 3D flight trajectories of the insects, we extracted aerial velocity and manoeuvring performance. The insects did not passively drift downwind and two of the three insects studied displayed distinct upwind flight behaviors. Maximal flight speed was slower than that of larger insects, but faster in terms of body length per second. The manoeuvrability of two out of the three studied species exceeded that of larger animals in terms of flight speed for a minimal turning radius. These findings advocate the inclusion of active flight in small insect dispersal models and suggest that miniaturization may result in improved aerial manoeuvrability.

## Introduction

Miniature insects, with wing and/or body lengths <1 mm, comprise the smallest actively flying animals. Their unique adaptations portray nature’s solutions to the problem of miniaturizing a complex flying animal to the size of some large unicellular organisms (Polilov 2015). Miniaturization should have detrimental effects for a flying animal. Large body, muscles, and wings are expected to enable faster locomotion compared to smaller animals (Bejan and Marden 2006; Tennekes 2009). Moreover, at the lower limits of insect body size, the dramatic decrease in size increases the effect of air viscosity on the flow around the insect. The phenomenon is characterized by lower Reynolds number (Re = body length × flight speed/kinematic viscosity of air). At a body length of 1 mm and flight speed below 1 m/s the Re of a flying insect is Re < 66. To compare, the Re for a fruit fly with a body length of ∼3 mm (Fernández-Moreno et al. 2007) flying at the same speed (1 m/s) is ∼200. A reduction in Re number from 200 to 66 leads to a 67% and 25% increase in the drag coefficient of smooth spheres and cylinders, respectively (Hoerner 1965). Furthermore, lower Re also leads to a decrease in the efficiency of aerofoils (McMasters and Henderson 1980). At the Reynolds number relevant to the wings of miniature insects (Re = wing chord length × air speed/kinematic viscosity of air, Re < 30, Sun 2023), air viscosity leads to a substantial increase in aerodynamic drag that resists the movement of the wings, combined with a reduction in the aerodynamic lift generated by the wings to keep the insect aloft (Horridge 1956; Ellington 1974). Consequently, studies focusing on dispersal tend to assume that miniature insects are weak flyers passively drifting in winds (Hardy and Milne 1938; Pasek 1988; Compton 2001; Kristensen et al. 2013; Strickland et al. 2017; Wainwright et al. 2017).

Air is a dynamically moving medium, requiring insects to orientate and move relative to their environment in winds of varying velocities (Chapman et al 2011). If the drag of miniature insects is proportionally higher, and their flight performance lower than that of larger insects, we might expect them to be incapable of compensating for even moderate wind velocities and drift downwind. Wind-borne transport can take the insects farther and faster compared to active flight upwind or in no wind conditions. Thrips have been observed moving through air without flapping, using their wings as parachutes to slow down their descent to the ground (Santhanakrishnan et al. 2014). It has also been suggested that the active flight of miniature insects is mostly vertical, aimed at keeping the insect aloft, while the wind disperses it horizontally (Taylor 1974; Wainwright et al. 2017; Wainwright et al. 2020). While drifting with the wind, miniature insects can control their transport by initiating and terminating flight, depending on environmental signals (Byrne 1999; Isaacs et al. 1999; Lopez-Reyes et al. 2023; Blackmer and Byrne 1993a). They can also alter the general drifting direction relative to the ground by actively flying in the air they are moving with toward visual targets or olfactory sources (Vosteen et al. 2020; Ben-Yakir and Carvalho 2021; Sarig and Ribak 2021; Ben-Yakir et al. 2023). Active flight in the upwind direction can also take place in the boundary layer close to the ground or within vegetation where wind speeds are much lower compared to conditions higher above ground (Taylor 1974; Sarig and Ribak 2021). Some release and recapture experiments have reported finding miniature insects upwind from the release site (Corbett and Rosenheim 1996; Simmons 2000; Desouhant et al. 2003). The mechanism that enabled these upwind transports remain obscure. We previously found that the miniature wasp *Eretmocerus mundus* (wing and body lengths < 0.7 mm) exposed to low-speed horizontal winds increased its horizontal flight speed to fly upwind toward a UV light source (Sarig and Ribak 2021). Other studies have reported a tendency of miniature thrips to fly upwind or control their downwind drift toward visual targets (Ben-Yakir and Carvalho 2021; Ben-Yakir et al. 2023). Therefore, active flight and its effect on drifting in winds should be accounted for when evaluating the dispersal potential of miniature insects at different wind speeds. Unfortunately, major knowledge gaps pertaining to constraints on flight performance are currently limiting such an attempt.

The few laboratory studies carried out to date suggest that the flight performance of miniature insects is in fact higher than expected for their small size (Isaacs et al. 1999; Farisenkov et al. 2020; Sarig and Ribak 2021). Miniature insects overcome the viscous flow that challenges aerodynamic lift production by utilizing “clap-and-fling” flapping kinematics that augments the unsteady lift beyond the expected steady values for these Reynolds numbers (Weis-Fogh 1973; Ellington 1974; Maxworthy 1979). They also harness the high drag generated on their wings to assist in propelling themselves through air (Cheng and Sun 2018; Farisenkov et al. 2022). With such unique adaptations for overcoming the impedance of low Re numbers, are small insects really limited in their flight performance compared to larger ones?

Active, controlled, free-flight involves manoeuvring. The ability to make a tight turn during flight requires a balance between body inertia, tending to keep the insect flying straight, and aerodynamic steering forces that generate the centripetal acceleration required for a curved trajectory. In simple circular motion, the required centripetal acceleration is inversely related to the turning radius and proportional to the square power of tangential (flight) velocity. Insects are highly manoeuvrable flyers (Dudley 2002; Fry et al. 2003; Bomphrey et al. 2009; Beatus et al. 2015; Dickinson and Muijres 2016; Sun et al. 2017; Roy et al. 2019). It is unclear how the low Re flow around the miniature wings and body affects the ability of small insects to generate large steering forces and torques. If smaller insects are weak flyers, then we would expect their manoeuvrability to be compromised as well. In contrast, if their flight performance is higher than expected for their body size, we may expect their manoeuvrability to exceed that of larger insects. To the best of our knowledge these alternatives have never been tested.

**Fig. 1:**
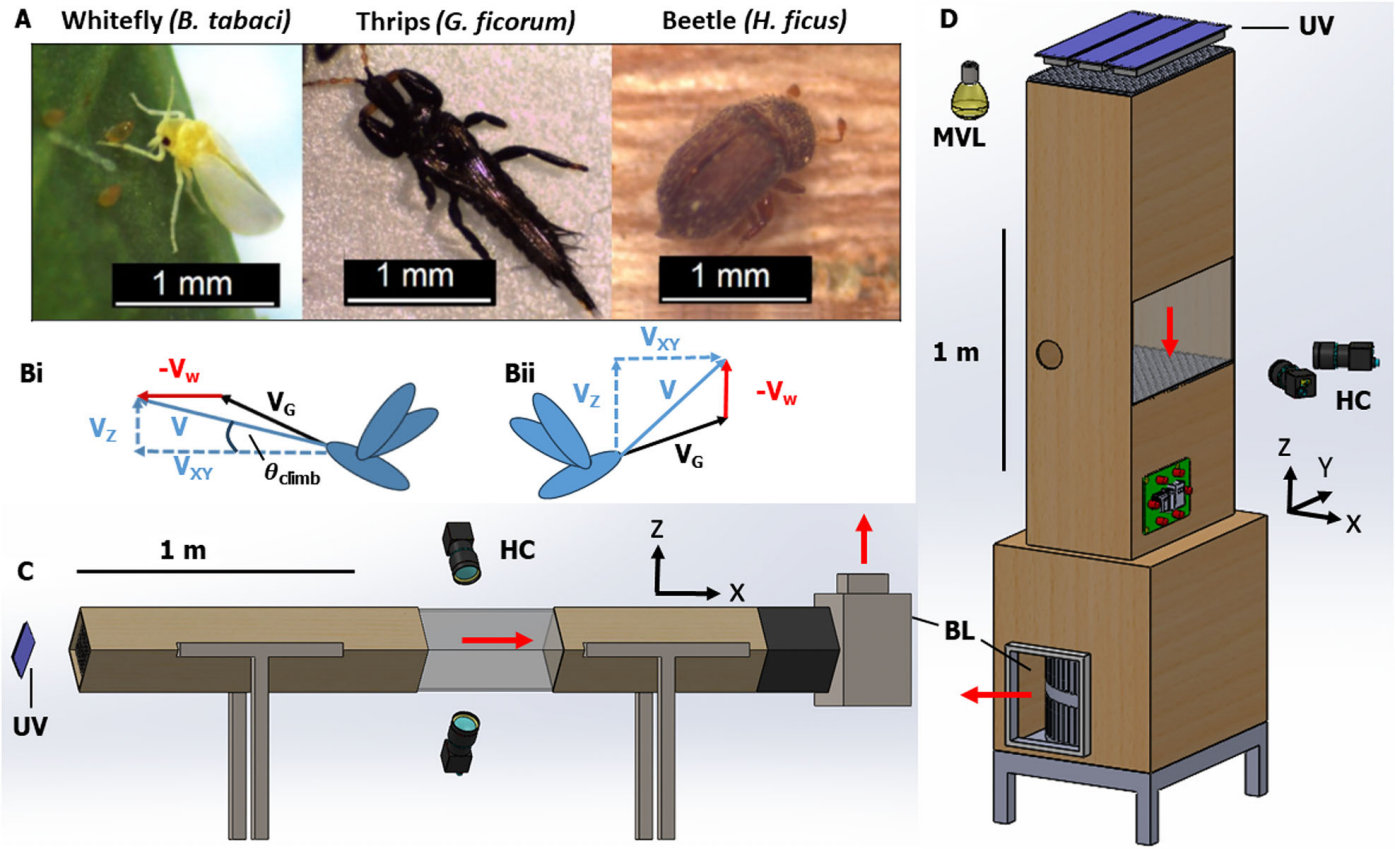
Methods. (A) The three insect species studied in the wind tunnels. The whitefly has a body length of 0.8 mm and wing length of 1 mm. The thrips has a body length of 2.2 mm and a wing length of 1 mm. The beetle has a body length of 1.3 mm and hindwing length of 1.8 mm. (B) vector addition of the 3D flight velocity relative to the ground (${V_G}$, black arrow) and wind velocity (${V_W}$, red arrow) gives the aerial speed of the insect (*V*, blue arrow) and its horizontal (${V_{XY}}$) and vertical (${V_Z}$) components in each video frame (dashes blue arrows). Bi and Bii show the addition of wind velocity in the horizontal and vertical wind tunnel, respectively. The horizontal (C) and vertical (D) wind tunnels used in the study. Red arrows denote the airflow in the tunnel. The flights of the insects are tracked at 3D using two synchronized high-speed video cameras. BL—Blower, HC—high-speed cameras, UV—ultra-violet light, MVL—mercury vapor lamp.

The vastly different physical and physiological constraints on flight at small size imply that what we know about the flight of larger insects may not hold for miniature ones. To bridge these knowledge gaps, we set to determine the flight performance and its relationship to flight behavior in winds in three small insects by tracking their free-flight inside a low-speed wind tunnel. The studied insects belong to three different insect orders with wing and/or body lengths smaller than 1.3 mm (See Fig. 1A for specific wing and body dimensions). Together they allow to test for shared challenges and converging solutions to the problem of flight at lower Re numbers in insects whose miniaturization evolved independently. Tests were done in both a horizontal and a vertical wind tunnel, with and without low-speed wind. From the tracked flight trajectories, we evaluated the limits to flight velocity and aerial manoeuvrability. We hypothesized: (1) that the three studied insects will be slower than larger insects (e.g., fruit fly size and larger, Supporting Information, SI1) and therefore less capable of overcoming winds; (2) that nonetheless, they actively fly in the presence of wind to control their transport rather than drifting passively downwind; (3) that manoeuvrability is improved in these insects compared to larger ones, due to inertial forces being attenuated by air viscosity at small size. The insights generated by the study can assist in modeling dispersal of small insects and expend our biomechanical understanding of natural flight toward the lower end of the insect size scale.

## Material and methods

### Animals

Whiteflies (*Bemisia tabaci*, Hemiptera, Fig. 1Ai) were collected with an aspirator from a greenhouse (see Ribak et al. 2016, for rearing conditions). Thrips, (*Gynaikothrips ficorum*, Thysanoptera, Fig. 1Aii) were collected from *Ficus benjamina* trees in Tel Aviv University campus (Central Israel). Bark beetles, (*Hypoborus ficus*, Coleoptera, Fig. 1Aiii), were collected from dry branches of *Ficus carica* trees at the western slopes of the Judaean Mountains (Central Israel).

### Set-up for the flight trials

The flight of the three species was studied once in a horizontal wind tunnel and again in a vertical wind tunnel. The horizontal wind tunnel (Fig. 1C) was previously described (Sarig and Ribak 2021). It has a cross-section of 0.2 × 0.21 m and a working section length of 0.5 m. An ultraviolet light source placed at the upstream end of the tunnel provided a stimulus for the insects to fly upwind. We placed the insects into a trimmed glass pipette positioned inside the working section of the wind tunnel. The pipette was tilted with its tip at the center of the working section and the base lower and downwind, to minimize flow interference at the tip (take-off point). The insects inside the pipette climbed up to the tip and exited through it one at a time. Outside they typically paused for a while before taking off voluntarily.

The vertical wind tunnel (Fig. 1D) was custom-built according to the design described in Blackmer and Phelan (1991) with several modifications: (1) It had a fixed rectangular cross-section of 0.45 × 0.75 m and a length (height) of 1.7 m. (2) A custom designed blower was installed at the bottom pulling the air out of the lower end of the tunnel. (3) The air entered the top of the tunnel through a series of 1/8 inch honeycombs (PAMG –XR1 5052; Plascore) that ensured laminar and uniform wind speed in the working section 1m beneath. We used a digital controller to adjust wind velocity in the working section by varying the DC voltage of the motor rotating the blower. The blower speed–wind speed relationship was calibrated using a hot-wire anemometer (SwemaAir40, Swema, Sweden). The homogeneity of the free-stream velocity in the working section was verified at the beginning of each experiment by moving the hotwire between various positions within the working section of the wind tunnel. To provide an incentive for the insects to fly upwind, a 32W UV light source (OMIK206-2 × 8W, Zhongshan Yaling Electric Appliance Co., China) was placed above the open upper end of the wind tunnel. Another mercury vapour lamp provided visible light. To prevent the mercury lamp from heating the air, it was mounted outside of the wind tunnel. The light emitted from the lamp was then reflected into the wind tunnel through a glass window and a 45° inclined mirror (Fig. 1B). The insects were placed in a small Petri dish placed at the center of the mesh (pore diameter 0.2 mm) comprising the bottom of the working section. The insect typically walked around the Petri dish edges for several minutes before flight commenced voluntarily. Experiments were carried out in room temperature 26°–29°C.

### High-speed recording

The flights of the insects in both wind tunnels were recorded by two synchronized high-speed cameras (Fastcam SA3, Photron Inc.) equipped with 85- or 28-mm (horizontal wind tunnel) or 50-mm (vertical wind tunnel) lenses (Nikkor, Nikon). Filming in the horizontal wind tunnel focused on the flight of the insect immediately after take-off (Supporting Information, Movie 01). Whiteflies are known to take-off by jumping with their wings closed. Wing opening and flapping starts only after the insect moved several body lengths in air (Ribak et al. 2016). Therefore, with the 85 mm lens the cameras were set to film at 2000 frames per second (FPS) and the flights were tracked from the first flapping cycle after take-off to the point when the animals exited the field of view of one of the two cameras. With the 28-mm lens, the cameras filmed at 250 FPS to track the insects longer through a larger field of view.

In the larger vertical wind tunnel, we tracked the insects flying over a much larger area (Supporting Information, Movie 02). The cameras filmed at 125 (thrips) and 60 (whiteflies and beetles) FPS and captured the flight from the take-off to the point where the insect exited the field of view or landed on the floor or walls of the wind tunnel. Each animal was tested only once, either in the horizontal or vertical wind tunnel. The cameras were spatially calibrated to extract 3D positions of landmarks observed in the two camera views using the DLTdv5 script (Hedrick 2008) for MATLAB (The MathWorks, Inc., USA). In the horizontal wind tunnel, we spatially calibrated the zoomed in cameras (85-mm lens) using the eight corners of a transparent cuboid object with known dimensions (1.94 × 1.24 × 1.24 cm) that was visible in the mutual field of view of both cameras (Hedrick 2008). In the zoomed out (28 mm) filming and in the vertical wind tunnel, we used a wand with two points fixed at a known distance apart to calibrate (Theriault et al. 2014) the mutual field of view of both cameras. The tracking volume in the vertical tunnel was 0.5 × 0.45 × 0.75 m. In both wind tunnels, the insect flight was tracked in a coordinate system where the + *Z*-axis is defined vertical and pointing up and the *X* and *Y*-axes are horizontal axes with the + *X*-axis pointing downwind in the horizontal wind tunnel (Fig. 1C, D). Because of the difference in tracking volumes, the mean recorded flight duration was 0.21 ± 0.255 s and 0.63 ± 0.528 s (mean ± SD, ∼40 and ∼120 flapping cycles) in the horizontal wind tunnel filmed with the 85 mm and 28 mm lenses, respectively, and 3.419 ± 5.153 seconds (>600 flapping cycles) in the vertical wind tunnel experiment. In both wind tunnels, some of the insects were recorded in stagnant air (the wind tunnel’s blower turned off) and some were recorded with the blower working to generate low wind speed. The wind speed used for each species was different (Table 1). It was found by trial and error in preliminary experiments where we gradually increased the wind speed and observed the take-off frequency. As the wind speed increased the insects became increasingly reluctant to take-off voluntarily (see the section “Discussion”). We therefore used the highest wind speed that still permitted multiple voluntary flights by the insects within a period of an hour.

**Table 1:** Sample size (number of tracked flights) for the three insect species and wind speed experiments.

Wind tunnel	Species	Wind speed (m/s)	*N* (trials)
Horizontal	*B. tabaci*	0	35
		0.13	22
		0.25	28
	*G. ficorum*	0	16
		0.12	23
		0.17	12
	*H. ficus*	0	13
		0.07	28
		0.14	14
Vertical	*B. tabaci*	0	30
		0.1	25
		0.2	22
	*G. ficorum*	0	25
		0.14	2
	*H. ficus*	0	20
		0.1	25

Each individual insect was tested once in one of the experiments (number of flight trials = number of tested insects).

### Data analysis

Position data from the two calibrated cameras were converted to 3D-flight trajectories by digitizing the insect’s head in each 2D video frame to extract the instantaneous 3D position of the animal. The raw positions data was smoothed by filtering with a Gaussian low-pass filter with a cut-off frequency of 10% the FPS to remove digitizing noise. Then, the instantaneous 3D velocity relative to the ground ($\mathop {{{\bf{V}}_{\bf{G}}}}\limits^{\rightarrow} $) was determined from a numerical time derivative of the smoothed data as in Rayner and Aldridge (1985). The aerial velocity ($\mathop V\limits^{\rightarrow} $) in each video frame was calculated by vector addition (Fig. 1B) of the instantaneous $\mathop {{{\bf{V}}_{\bf{G}}}}\limits^{\rightarrow} $ in that frame with the (-) wind velocity in the wind tunnel ($\mathop {{V_W}}\limits^{\rightarrow} $):


(1) \begin{eqnarray*}
\mathop V\limits^{\rightarrow} = \mathop {{{\bf{V}}_{\bf{G}}}}\limits^{\rightarrow} + \left( { - \mathop {{V_W}}\limits^{\rightarrow} } \right)
\end{eqnarray*}

**Fig. 2:**
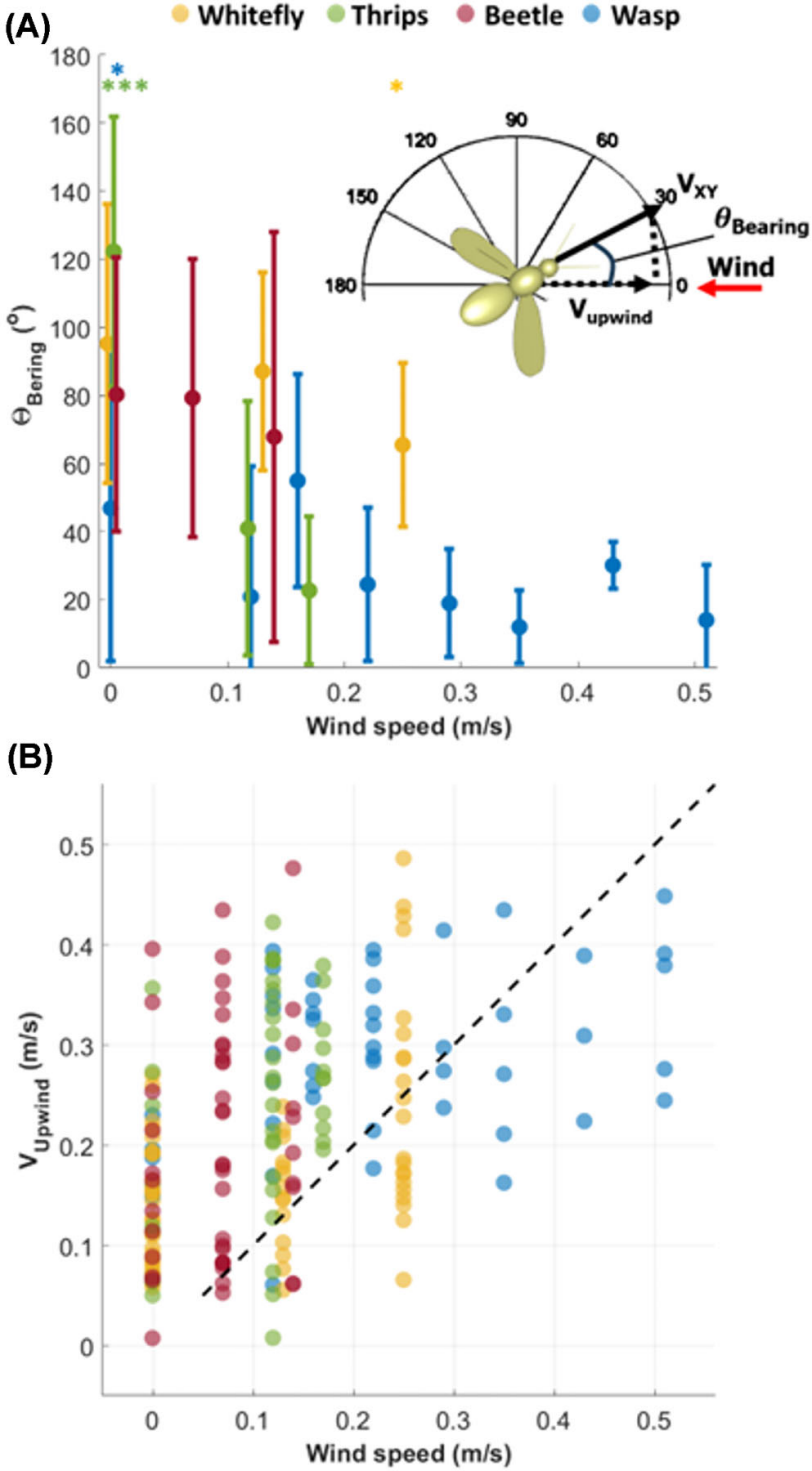
Flight in the upwind direction as a function of wind speed in miniature insects from four taxonomic groups. The horizontal flight direction relative to the wind direction ($\theta $_bearing_) and the upwind flight speed (${V_{\textit{upwind}}}$) are defined in the inset depicting the insect from above. (A) Median $\theta $_bearing_ was variable and approximately 90° (across the wind tunnel) when the wind was off (wind speed = 0) and decreased to fly in the upwind direction in whiteflies, wasps, and thrips as the wind was turned on or increased. The “wasp” data refer to *Eretmocerus mundus* studied previously in the same horizontal wind tunnel (Sarig and Ribak 2021). Circles denote the median and the error bars are ± 1 standard deviation of trials at different wind speeds. Colors denote insect species. Asterisks denote significant difference between the “zero wind” category and other wind groups in post-hoc tests for the thrips and wasp, and difference of the 0.25 m/s wind category from the zero wind and 0.13 m/s wind categories in whiteflies (*P < 0.05, ***P < 0.001). (B) Using the same color code, each circle represents the mean upwind flight speed (${V_{\textit{upwind}}}$) of flight sections in which the insect flew in the upwind direction ($\theta $_bearing_ < 45°). The dashed line has a slope of 1 and intersect = 0. Data points below the line are insects that had a downwind displacement relative to the ground despite flying in the upwind direction. Insect above the dashed line make upwind displacement relative to the ground in head wind.

In the horizontal wind tunnel, the instantaneous horizontal flight angle relative to the wind direction in each video frame was calculated from the horizontal (${V_X},\,\,{V_Y})$ components of $\mathop V\limits^{\rightarrow} $:


(2) \begin{eqnarray*}
{\theta _{\textit{Bearing}}} = {\tan ^{ - 1}}({V_Y}/{V_X})
\end{eqnarray*}

while accounting for the sign of ${V_X}$ and ${V_Y}$ so that $0^\circ \le {\theta _{\textit{Bearing}}} \le 180^\circ $ where flight upwind is defined as ${\theta _{\textit{Bearing}}} = $ 0° and flight in the downwind direction is defined as ${\theta _{\textit{Bearing}}} = $ 180° (see inset in Fig. 2A). For the analysis of upwind flight, we isolated sections within flights in the horizontal wind tunnel where the flight direction in the horizontal plane was ${\theta _{\textit{Bearing}}}$<45°. Then, the instantaneous aerial flight speed along the *X*-axis of the isolated data (${V_{\textit{upwind}}}$) was averaged for each individual and plotted (Fig. 2B) against the free stream velocity in the wind tunnel. A line with a slope of 1.0 passing through the origin divided the data into cases in which the animals flew faster (data above the line) and slower (data below the line) than the head wind. The distinction implies net upwind displacement relative to the ground in the former as opposed to insects that were flying in the upwind direction through air but drifting backward (having downwind displacement) relative to ground in the latter. Figure 2 includes data on the flight of a fourth species (“Wasp”), the miniature wasp (*Eretmocerus mundus*) measured in the same horizontal wind tunnel in a previous study (Sarig and Ribak 2021).

In both wind tunnels, the instantaneous climb angle in each video frame was calculated from the horizontal (${V_{XY}})$ and vertical (${V_Z}$) components of $\mathop V\limits^{\rightarrow} $:


(3) \begin{eqnarray*}
{\theta _{\textit{climb}}} = {\tan ^{ - 1}}({V_Z}/{V_{XY}}),
\end{eqnarray*}

Where instantaneous ${V_{XY}}$ was calculated as:


(4) \begin{eqnarray*}
{V_{XY}} = \sqrt {V_X^2 + V_Y^2}
\end{eqnarray*}

${\theta _{\textit{climb}}}$ is the flight direction in the vertical plane, ${V_Z}$ is the insect’s climbing rate (Fig 1B).

**Fig. 3:**
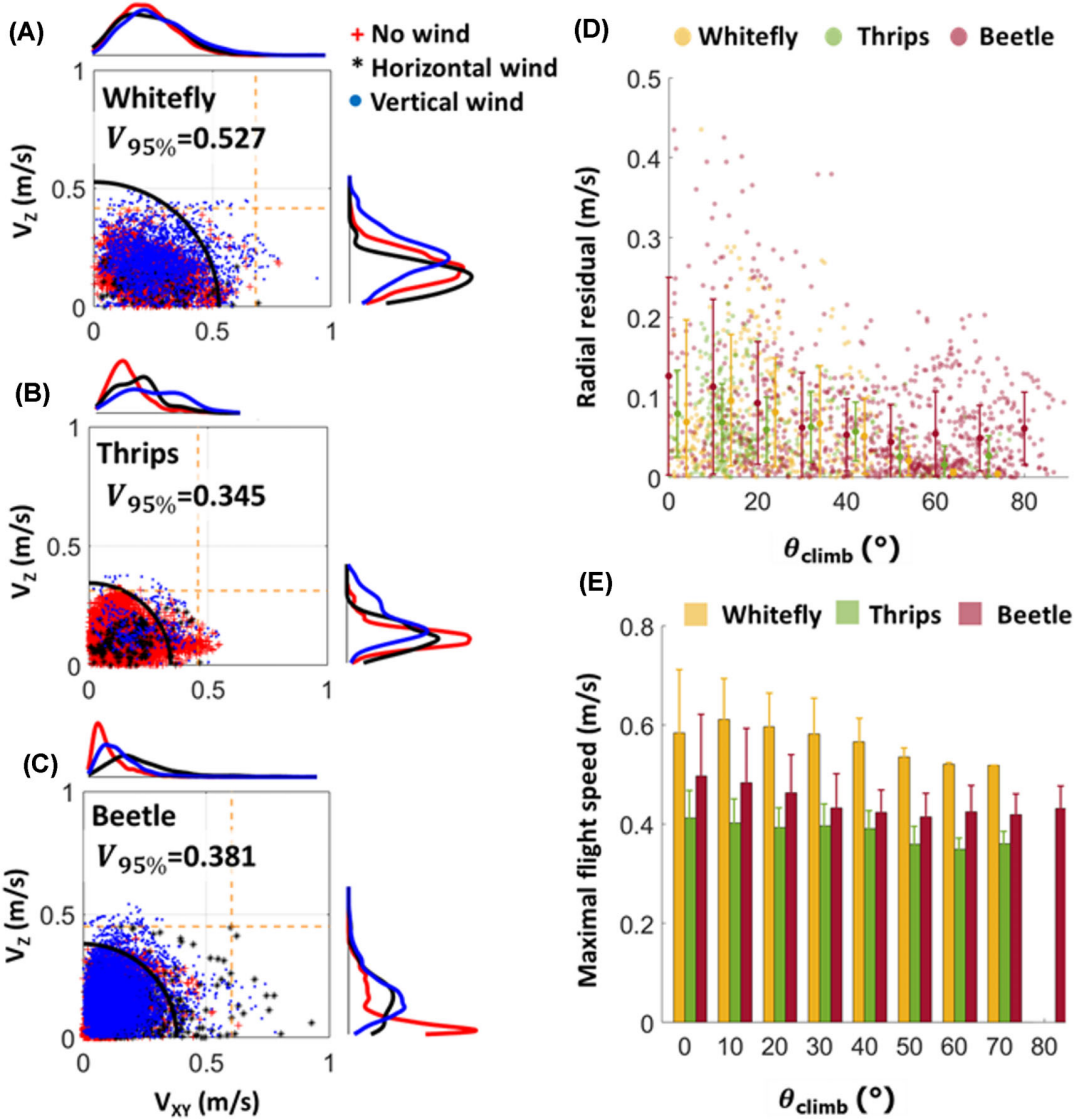
Flight performance envelope. (A–C) Instantaneous horizontal (${V_{XY}}$) versus vertical (${V_Z}$) aerial flight speeds from all trials in the current study. Red crosses, black asterisks, and blue dots denote experiments without wind, with horizontal wind, and with vertical wind, respectively. The distribution of the data along each axis is shown outside the plot area using the same color code. V_95%_ is the 95th percentile of the 3D flight speed for each insect and the black quarter-circle denotes the projections of V_95%_ for hypothetical climb angles ranging from 0° to 90°. Orange dashed lines denote the 95th-precentile of ${V_{XY}}$ and ${V_Z}$ calculated separately from all the data of each species. (D) The distribution of the data points outside the quarter circles in A–C as a function of climb angle. The vertical axis is the radial distance of points from the upper 5% of flight velocity from the species-specific V_95%_ perimeter. Dots represent individual instantaneous measurements. Large circles and error bars represent the mean ± 1 standard deviation of data binned into 10-degree intervals of climb angle. (E) The maximal flight speed for each climb angle category. Colors in D and E correspond to the three insect species according to the legend in E.

For identifying the flight performance limits, all data with a downward flight direction (${\theta _{\textit{climb}}}$<0) were excluded from the analysis. We used the upper 5% of the observed $\mathop V\limits^{\rightarrow} $ data, but accounted for the effect of ${\theta _{\textit{climb}}}$ on maximal flight speed. Specifically, we found the 95% percentile of the 3D speed |$\mathop V\limits^{\rightarrow} $| for each species (V_95%_) and plotted its vertical and horizontal components for hypothetical climb angles 0°$\le {\theta _{\textit{climb}}} \le 90^\circ $ on a ${V_Z}$ as a function of ${V_{XY}}$ plot. The resulting arc encompassed most of the observed velocity data of the species (Fig 3). Data points outside the arc were scored by their radial distance from the arc. We then binned these radial distances into nine climb angle bins (each bin 10° wide, Fig. 3D) and averaged the data in each bin. This mean of each bin + V_95%_ was defined as our performance limit for that climb angle (Fig. 3E).

The instantaneous curvature of the trajectory (${k_{XY}})$ in the horizontal plane was calculated as:


(5) \begin{eqnarray*}
{k_{xy}} = \left| {\left( {{V_X}{a_Y} - {V_Y}{a_X}} \right)} \right|/{\left( {V_X^2 + V_Y^2} \right)^{3/2}},
\end{eqnarray*}

Where $\mathop a\limits^{\rightarrow} $ is the instantaneous acceleration vector calculated from the numerical time derivative of $\mathop V\limits^{\rightarrow} $ as in Rayner and Aldridge (1985).

Since the curvature is the inverse of the turning radius, the instantaneous centripetal acceleration required for the curved trajectory in the horizontal plane was calculated for each video frame as:


(6) \begin{eqnarray*}
{a_C} = {k_{xy}} \cdot {V_{XY}}^2
\end{eqnarray*}

Velocity and acceleration derived numerically from high-speed movies tend to be noisy even after smoothing the positions data. To: (1) mitigate the amplification of noise by the numerical differentiation, (2) prevent bias from outliers, and (3) equalize camera filming rate effects across experiments, we low-passed the instantaneous data of flights recorded at a video frame rate > 60 FPS. This was achieved by binning the data of each flight into discrete consecutive time intervals of 16.67 ms (60 Hz sampling rate) and using the median value of all instantaneous data (video frames) within a bin as its representative value. This binning procedure yields more conservative estimates of flight performance than standard smoothing techniques such as moving averages or frequency filters that may preserve artificial data peaks. The conservative measures were taken to ensure the reported flight envelope represents biologically meaningful performance rather than tracking noise.

For comparing manoeuvrability, we limited our analysis to horizontal maneuvers within the trajectories. A maneuver was defined as any continuous flight section within the trajectory that lasted $\ge $ 33.34 ms, while maintaining ${V_{XY}}$>0.1 m/s and a turning radius of $0.1 < {k_{xy}}^{ - 1} < 100$ mm (e.g., Supporting Information, SI2, Movie 03). Each maneuver was considered as a statistically independent observation, although most trajectories did include more than one maneuvers. In total, we analyzed 1227 maneuvers from 177 individual insects (Supporting Information, SI3). The upper boundary for turning performance was defined by taking all the manoeuvring data for each species (including data for other animals, Supporting Information, SI3), finding the median flight speed and median turning radius for each maneuver, then plotting these medians from all maneuvers on a flight speed versus turning radius plot and tracing the maximum velocity data for each increment along the horizontal axis (turning radius).

### Statistical analysis

**Fig. 4:**
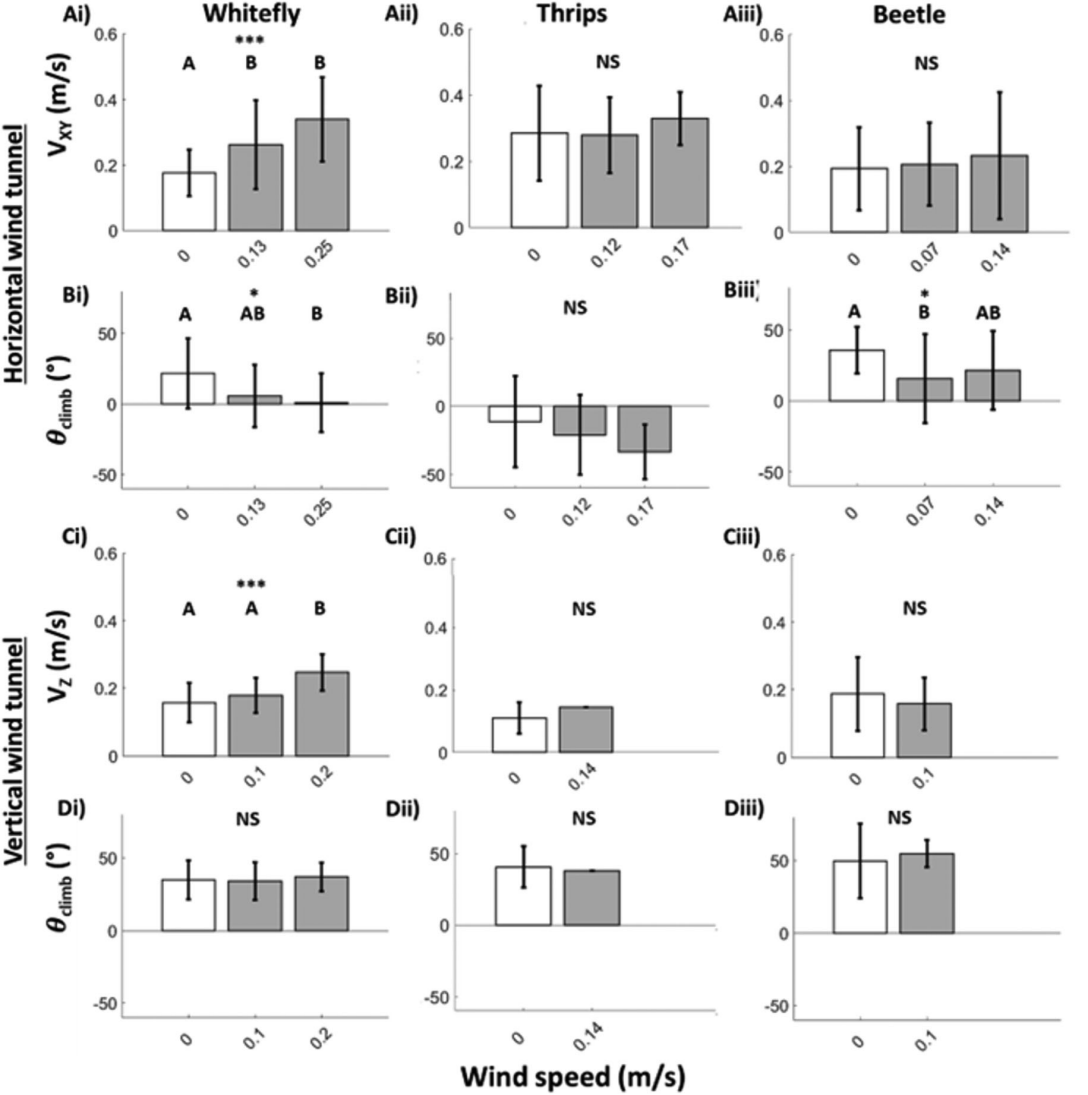
Wind effect on flight. Median flight speed (A, C) and climb angles (B, D) measured in the horizontal (A, B) and vertical (C, D) wind tunnels. Empty bars denote flights when the wind was off (stagnant air), full bars denote that the wind was on. The three columns in each panel correspond to whiteflies (i), thrips (ii), and beetles (iii). Error bars denote ± 1 standard deviation. Note that (A) depicts the horizontal flight speed (${V_{XY}}$) while (C) depicts the vertical flight speed (${V_Z}$). Asterisks denote statistical significance: *(*P* < 0.05), **(*P* < 0.01) and ***(*P* < 0.001). NS—no significance (*P* > 0.05). Letters A and B denote statistical similarity according to post-hoc tests.

In total, we recorded flight trajectories from 340 insects as detailed in Table 1. To compare flight velocity across wind speeds (Fig. 4), each tracked trajectory (hereafter “a trial”) is represented in the statistical analysis by its median of instantaneous flight speed and climb angle. We analyzed the data for each insect species separately. To test for downward air flow effect (present/absent) on the dependent variables in the vertical wind tunnel we used the *t*-tests, when the data were normally distributed and Mann–Whitney U tests when normal distribution could not be assumed. Normal distribution was tested using Shapiro–Wilkinson test. Similarly, linear correlations were tested using Pearson’s or Spearman’s rank correlation coefficients depending on normality. In the horizontal wind tunnel, and in the whiteflies tested in the vertical wind tunnel, each species was tested in more than one wind speed. Thus, we used one-way ANOVA with unequal N HSD post-hoc tests for normally distributed data. Kruskal–Wallis ANOVA followed by multiple comparisons post-hoc tests were used if normal distribution could not be assumed. In both cases, wind speed was the factor and wind speed = 0 (no wind) was one of its levels. In the trajectories recorded in the horizontal wind tunnel with the 28 mm lens, we compared the ability of the insects to make progress upwind relative to the ground by subtracting their position along the *X*-axis at the take-off point from the position at the trajectory end. We compared this measured distance with the distance expected for passively drifting downwind using the Wilcoxon matched pair test. All analyses were carried out in STATISTICA v. 12 and SPSS.

## Results

### Response to horizontal wind

Whiteflies and thrips responded to light horizontal wind by changing their median flight direction in the horizontal plane (${\theta _{\textit{Bearing}}})\,\,$to a more upwind flight direction compared to the no wind condition (Fig. 2A, ANOVA F_(2,82)_ = 5.44, *P* = 0.006, Kruskal–Wallis ANOVA H_(2,51)_ = 22.85, *P* < 0.001, respectively). The beetles did not change flight direction due to the wind (Kruskal–Wallis ANOVA F_(2,55)_ = 0.05, *P* = 0.97), and their flight did not have a specific direction with respect to the wind direction. When only the upwind flight sections were analyzed, some insects from all tested species flew at ${V_{\textit{upwind}}}$ (Fig. 2A) speeds lower than the wind speed, thus flying in the upwind direction but having a downwind displacement relative to the ground (Fig. 2B). The remaining insects flew faster than the light wind, making progress upwind relative to the ground.

In the horizontal wind, whiteflies increased their horizontal aerial flight speed (${V_{XY}}$, Kruskal–Wallis ANOVA, H_(2,85)_ = 29.06, *P* < 0.001). With the flight speed increasing in both wind speeds compared to the no wind condition (Fig. 4A). The thrips and beetles did not increase their horizontal flight speed in wind (thrips: ANOVA F_(2,48)_ = 1.36, *P* = 0.267; beetle: Kruskal–Wallis ANOVA, H_(2,55)_ = 2.56, *P* = 0.278).

The climb angle was significantly lower in whiteflies (ANOVA, F_(2,82)_ = 4.7, *P* = 0.012) and beetles (F_(2,52)_ = 3.37, *P* = 0.042) in wind compared to the no wind condition, reflecting a more horizontal flight in wind condition. In contrast, the median climb angle of the thrips was negative and did not change between wind and stagnant air conditions (Kruskal–Wallis ANOVA H_(2,51)_ = 4.84, *P* = 0.109; Fig. 4B).

By the end of the longer flight trials in the horizontal wind tunnel, some of the insects were positioned downwind of their take-off point, but all three species were significantly upwind relative to their expected position for passively drifting downwind (Wilcoxon signed-rank test, whiteflies: *P* < 0.001; thrips: *P* = 0.028; Beetles *P* = 0.017). The mean upwind discrepancies from the expected position were 10.7 ± 11.3, 6 ± 8.3 and 13.5 ± 13.6 cm in the whiteflies, thrips and beetles, respectively (Supporting Information, SI4).

### Effect of vertical wind on climb angle and vertical flight speed

Downward air flow in the vertical wind tunnel resulted in increased climbing rate (${V_Z}$) in whiteflies compared to the no wind condition (ANOVA F_(2,74)_ = 17.27, *P* < 0.001, Fig. 4C). The beetles did not display a similar response to vertical air flow (t_(43)_ = −1.34, *P* = 0.188). The vertical air flow had no effect on the climb angle of any of the species (Fig. 4D, whiteflies: ANOVA F_(2,74)_ = 1.98, *P* = 0.146; beetles: Mann–Whitney U = 206, *P* = 0.32). The thrips were extremely reluctant to take-off in downward directed wind. Out of dozens of insects introduced to the tunnel for hours, only two took off in vertical wind (Table 1) and both flew at low climb rates (Fig. 4Cii), leading to very short flights that ended in the insects reaching the floor.

### Limits to flight velocity

Figure 3 combines the data from both wind tunnels to visualize the flight performance envelop of the three insects. The whiteflies and beetles had a 95% percentile of climbing rate (${V_Z})$ slightly below 0.5 m/s, while thrips were confined to ${V_Z}$<0.32 m/s. In whiteflies and beetles, the 95th-percentile of ${V_{XY}}$ was 0.68 and 0.60 m/s, respectively, while in thrips it was 0.46 m/s. Thus, in all three species top horizontal speed exceeded the top climbing rate (Fig. 3A–C). The 3D flight speed ($| V |$) at the performance limit showed a significant decline with increasing climb angle (Fig. 3E, Spearman rank, whiteflies: r = −0.93, *P* = 0.002; thrips: *r* = −0.90, *P* = 0.005; beetles: *r* = −0.75, *P* = 0.026).

### Limits to manoeuvrability

**Fig. 5:**
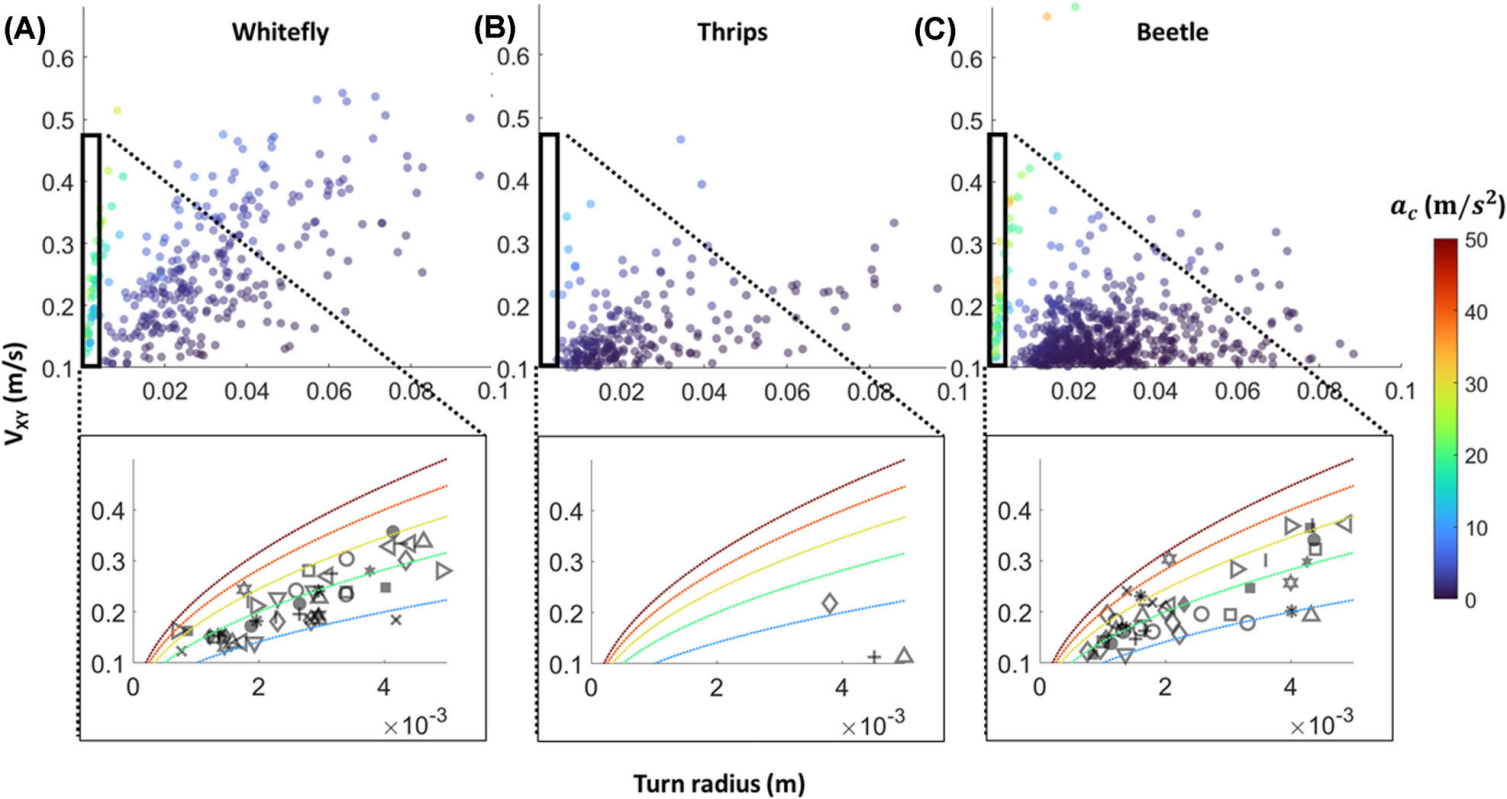
Turning performance. Each symbol represents a maneuver. In the upper panel, the color of the dots denotes the median centripetal acceleration (${a_c})\,\,$of the maneuver according to the colorbar on the right. (A–C) correspond to Whitefly, Thrips, and Beetle data, respectively. Black rectangles mark the area corresponding to maneuvers with the smallest turning radius ($\le $5 mm), which are magnified in the lower panel with different symbols for each individual insect. The color-coded contours denote the centripetal acceleration using the same colorbar.

While manoeuvring, whiteflies displayed the highest centripetal acceleration across all measured maneuvers (maximal ${a_C}$= 36 m/s^2^) closely followed by the beetles (maximal ${a_C}$= 35 m/s^2^) and the thrips reached a lower maximal ${a_C}$= 13 m/s^2^ (Fig. 5).

## Discussion

Very small insects face mechanical constraints associated with flight at Re numbers in which aerodynamic lift production is limited and viscous drag is high. We hypothesized that the small insects studied here: (1) are slower than larger insects but fly faster than expected for their small size, (2) will actively fly in wind conditions to control their flight direction, and (3) have improved manoeuvrability compared to larger insects. At the maximum flight speeds reported here the Re number for the body of the three insect species were 37, 67, and 50 for the whiteflies, thrips, and beetles, respectively. These numbers are at least threefold lower than the Re numbers for fruit flies and two orders of magnitude lower than the Re of some larger insects (Supporting Information, SI1). Despite of the expected decrease in flight performance with decreased Re number, the three insect species displayed active flight and high manoeuvrability combined with a tendency to fly in the upwind direction, rather than passively drifting downwind. Therefore, we found support to all three hypotheses as explained below.

### Top flight speed

**Fig. 6:**
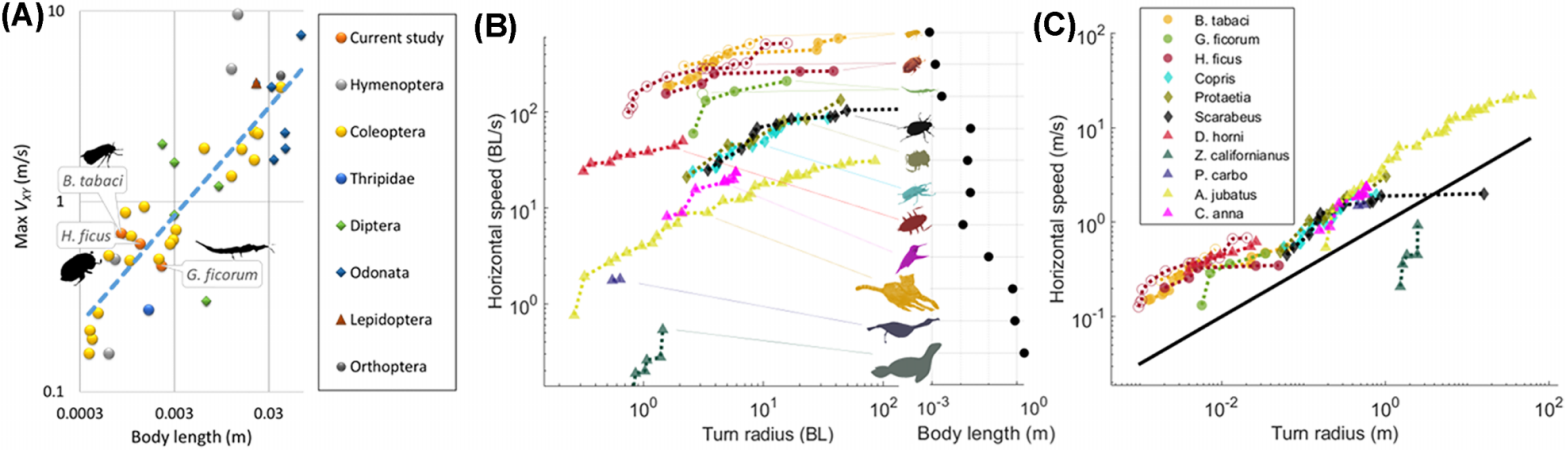
Flight performance of miniature insects compared to larger animals. (A) The relationship between maximal flight speed and body length. The insects from the current study are labeled. Other symbol shapes and colors correspond to data on other insects from the literature (Supporting Information, SI1) and taxonomic affiliation. Dashed line shows the allometric relationship ($U = 23.075B{L^{0.57}}$) of the depicted literature data (*r* = 0.85, *P* < 0.001, *N* = 37 species) where $U$ is the maximal flight speed in m/s and BL is body length in m. (B) The relationship between turning radius and speed (both divided by body length, BL) for various animals varying in body length over three orders of magnitude (right panel). Data for each species correspond to the maximal speed at minimal radius from several turns (see the section “Material and methods”). Symbols denote the different data sources, where circles are data from the current study (for the three insects) with full circles denoting experiments with horizontal wind and empty circles denoting experiments without horizontal wind. (C) Same as (B), except that the units are m and m/s. The black solid line represents $U = \sqrt r $ predicted from circular motion. The cheetah’s maneuvering is under different biomechanical constraints than in swimming and flying animals. It is included here as the fastest terrestrial runner simply to provide perspective on performance.

While there are data on flight speed for a wide range of larger insects (Supporting Information, SI1), information on the top flight speed of miniature insects is limited to relatively few studies and insect groups (Farisenkov et al. 2020; Sarig and Ribak 2021; Sun 2023). Figure 6A combines data from these studies with data from the current one (95th percentile of ${V_{XY}}$) as well as published data on top flight speeds of some lager insects. The allometric relationship of the published data spanning two orders of magnitude in body size (excluding the three species studied here) is: $FS = 23.08B{L^{0.57}}$ where FS and BL are flight speed (in m/s) and body length (in m), respectively. The whiteflies are positioned above this mean trend line indicating above average flight performance for similarly sized insects. Published data on the miniature wasp *E. mundus* studied in the same wind tunnel (body length 0.7 mm, top flight speed 0.5 m/s, Re = 23, Sarig and Ribak 2021) also falls above the regression line while the slightly larger beetles and thrips fall on and below the line, indicating average and below average speeds for similarly sized insects, respectively. The exponent of the allometric relationship including the three species studied here (0.45, 95% confidence interval ± 0.141) is significantly smaller than unity. It supports the notion that although flight speed diminishes with a decrease in insect size, in terms of body length per second our insects have higher flight speeds than the larger ones shown in Fig 6A. The body length–flight speed relationship shown in Fig. 6A encompasses insects from different taxonomic orders measured at vastly different experimental conditions (See Table S1 for methodological differences). Our study that used the same research setup on four very different insects clearly shows differences in flight performance between species. Therefore, the trend in Fig. 6A cannot describe a purely physical scaling effect (which might be better identified within each insect order). Nevertheless, the trend corroborates previous reports (Farisenkov et al. 2020) and our hypothesis that small insects fly faster relative to their size than larger ones that fly at higher Re numbers and are presumed to be less impeded by air viscosity. Bejan and Marden (2006) proposed that flight speed should increase with body mass to the power of 1/6 (i.e., $V \propto {m^{{{1} \!/ \!{6}}}})$ at the body mass range of flying animals. The mass of an animal is roughly proportional to its volume (assuming similar body densities), i.e., $m \propto {L^3}$, where $L$ is body length. Therefore, we can expect from Bejan and Marden’s (2006) relationship $V \propto {L^{{{1} \!/ \!{2}}}}$. The exponent of the relationship shown in Fig. 6A is not significantly different than 0.5. If the drag associated with lower Re number was a dominant effect impeding flight, we would expect a focus on the lower end of the flight speed–body length relationship to yield an allometric exponent $\gg $0.5, implying that smaller insects are flying slower than expected for their size. Hence, we find no support to the idea of reduced flight performance at lower Re numbers. Miniature insects may compensate for the decrease in their Re number by flying faster than expected for their size, thus, escaping the impediment of even lower Re number. Another possible explanation for the high maximal flight speeds is the need to overcome mild air currents in the natural environment to enable controlled flight toward visible or olfactory targets. These two explanations are not mutually exclusive and both suggest that miniature insects have high power outputs for their size, that likely come at high energetic flight costs (Farisenkov et al. 2022; Sarig et al. 2025).

It is difficult to estimate the maximal flight speed of an insect since free-flying insects spend most of the time flying at submaximal performance level. Here, using the 95th-percentile of observed ${V_{XY}}$ to represent top flight speed in Fig. 6A was a compromise between selecting a higher percentile, increasing the risk of overestimation due to inclusion of outliers; and selecting a lower percentile risking an underestimation. Since our hypothesis was that the studied species are faster than expected we preferred a conservative estimate of top flight speed that would allow higher confidence to accept this hypothesis. To identify the limits of the flight performance envelop, we had to account for the relationship between maximal flight speed and climb angle. To do so, the upper 5% of the flight velocity data were used. Here as well, the procedure followed data smoothing, binning, and filtering to a sampling rate of 60 Hz. That procedure attenuated the effect of extreme values to ensure that the 5% upper values used here are conservative estimates and not outliers from the tracking noise. The observation that the upper limit to flight speed decreased with increasing climb angle (Fig. 3E) likely reflects the elevated power required for ascending against gravity and/or a higher drag on the body when flying vertically (see below). Either way, it suggests that in the upper 5% of flight velocity the insects indeed flew at, or close to their maximal power limit. Thus, the conservative underestimation of the flight performance limits was probably slight relative to the actual values.

### Climb angle

Climb angle and climb rate (eq. 3) are limited not only by the drag on the body of the flyer but also by their effect on the lift-based propulsion of flapping and revolving wings used to support the body in air (Bramwell et al. 2001; Meng et al. 2017; Liu and Sun 2024). Because faster climbing requires additional power, power availability limits flight speed for a given climb angle (Liu and Sun 2024). The negative relationship between flight speed and climb angle observed here suggests that miniature insects conform to a similar power limit on climb rate, despite their unique propulsion involving “clap-and-fling” (Weis-Fogh 1973) and the drag on their wings (Weis-Fogh 1975; Cheng and Sun 2018, 2021; Farisenkov et al. 2022). However, the current analysis based on flight trajectories lacks data on body pitch, flapping frequency, and wingbeat kinematics, preventing the determination of the mechanism by which climb angle affected 3D flight speed in our insects.

Downward air flow in the vertical wind tunnel forces insects to increase climb rate in order to maintain level flight relative to the ground. The whiteflies increased their climbing rate indicating that they compensated for downdraft. However, they did not concurrently increase their climb angle, signifying that they increased their horizontal flight speed as well. Blackmer and Byrne (1993b) reported extremely prolonged vertical flights (between 10 min and 2.5 h) in whiteflies flying in a vertical wind tunnel, albeit this phenomenon was limited to few specific individuals (termed “long flyers”) out of the larger population. The vertical wind speeds they reported were manually controlled during flight and reached up to 1 m/s. In our constant low (<0.2 m/s) vertical wind speeds, we did not measure climbing rates that can explain vertical flights at speed > 0.5 m/s (Figs. 3, 4). We also did not observe prolonged vertical flights. The whiteflies were reluctant to fly at a climb angle of 90°, and with a mean climb angle of ∼45° (Fig. 4) most of the insects landed on either the floor or walls of the wind tunnel after a few seconds. The source of this discrepancy from Blackmer and Byrnes' (1993b) results is unclear. It may be due to our lower sample size or due to the differences in the experiment set-up (detailed in the section “Material and methods”).

Judging by their climb angles and inconsistent flight direction, the beetles displayed a higher tendency for vertical flights (Fig. 3C, E) but they did not change their flight behavior in the presence of vertical wind to compensate for the downdraft (Fig. 4C, D). In thrips, the reported use of parachuting (Santhanakrishnan et al. 2014) as a passive transport strategy may explain their preference not to take-off in wind with a downward directed velocity component. Notably, the maximum climb rate of the thrips observed in this study exceeded the vertical wind speed in the vertical wind tunnel (Fig. 3B). Therefore, the thrips reluctance to fly vertically against a downdraft was not due to a physiological or biomechanical limitation.

### Manoeuvrability

We hypothesized that the higher effect of air viscosity associated with the reduction in body size would improve the aerial manoeuvrability of our insects. Figure 6B compares the turning performance observed here with published data for other running, swimming, and flying animals executing extreme maneuvers. When both the flight speed and turning radius are normalized by body size the three miniature insect species have the highest flight speed for a given turning radius, with the whiteflies having the highest manoeuvrability followed by the bark beetles and then the thrips (Fig. 6B). Thrips were reluctant to make tight (radius < 5 mm) turns in our study and despite their lower centripetal accelerations (Fig. 5) their manoeuvrability still outperforms larger animals in proportion to their size. In terms of actual flight speed and turning radius, the relationship between the two variables (on a logarithmic scale) shares a similar slope with the rest of the (larger) turning animals (Fig. 6C). But the bark beetle and whitefly seem to have a higher intersect, suggesting that they are capable of taking tight turns at faster speed compared to larger animals. The thrips with their reduced flight and manoeuvring performance seem to continue the relationship between flight speed and turning radius observed in the larger animals. The superior manoeuvring capability of the whiteflies and beetles holds regardless of the flight speed used being the aerial or ground speed, as evident by comparing the data with and without wind conditions in Fig. 6C. Performing tight maneuvers at high speed requires high centripetal accelerations. Flying animals and airplanes achieve this by making banked turns in which the dorsal side of the body is rolled toward the turn center. This rolling shifts the lift vector from the wing to have a large component toward the turn’s center, providing the needed radial centripetal force. It is currently unclear how miniature insects with their reduced lift, due to lower Re (Ellington 1974; Horridge 1956), achieve this task. Miniature insects may use wing drag rather than lift for generating the side forces and turning torques needed. Therefore, they may benefit from the higher wing drag associated with more viscous flow (Jones et al. 2015; Cheng and Sun 2018; Farisenkov et al. 2022).

A more plausible, but not mutually exclusive option is geometrical. Smaller animals may take advantage of their higher ratio of surface area to volume. Since $m \propto {L^3}$ (explained above), whereas the aerodynamic forces that are used by the animal to steer are proportional to wing area ($\propto {L^2}$). The centripetal acceleration, ${a_c} = \frac{{{F_c}}}{m}$ is proportional to $\frac{{{L^2}}}{{{L^3}}} \propto {L^{ - 1}}$ implying higher accelerations for smaller animals. Similarly, If the mass moment of inertia ($I$) is simply scaled with body length according to geometrical similarity ($I \propto {L^5}$) and similarly aerodynamic torque ($\tau \propto {L^3}$), then a smaller body may be advantageous for generating both linear and angular accelerations ($\frac{F}{m} \propto {L^{ - 1}}$ and $\frac{\tau }{I} \propto {L^{ - 2}}$, respectively). This geometrical scaling argument knowingly overlooks variance in flapping frequency, wing shape, and flapping kinematics that determine aerodynamic force and torque generation. However, some support to this scaling argument can be found in a recent computational study that showed that the longitudinal instability, that is inherent to hovering insects, increases as body size decreases due to the lower mass failing to dampen small disturbances in their flight (Lyu and Sun 2022; Sun 2023). This higher instability in smaller insects is caused by the same decrease in body inertia relative to fluid forces. We propose that in addition to geometrical scaling, the lower Re number of the smallest insects adds to a greater reduction in body inertia in proportion to drag on the body, facilitating making tighter turns. Therefore, higher manoeuvrability in miniature insects may not be an adaptation due to their lifestyle but rather an inherent consequence of smaller size.

### Response to horizontal wind

The whiteflies and thrips showed a tendency to fly upwind in the horizontal wind tunnel when light horizontal winds were present. Although the beetles did not display a distinct behavioral response to horizontal wind, their flights trajectories included sections of upwind flight as well. Flight in the upwind direction occurred in the three species even if the flight speed was lower than the wind speed resulting in downwind displacement relative to the ground of some of the insect. Active flight in the upwind direction even while drifting downwind relative to the ground, has been reported before in *E. mundus* (Sarig and Ribak 2021; Fig. 2), amounting to four taxonomically distinct groups of small insects sharing the tendency to fly in the upwind direction. Preference for flight upwind even while drifting downwind may serve olfaction. In upwind flight, the insect encounters molecules coming from the flight direction, whereas in downwind flights the airborne molecules encountered during flight do not contribute to reaching the source. Vosteen et al. (2020) found that the host finding efficiency of a parasitic wasp (*Cotesia glomerata*) was reduced when flying downwind compared to flying upwind. The authors attributed this difference to the availability of herbivore-induced plant volatiles near the infested plant.

In two out of the three species (whiteflies and beetles), we found a decrease in climb angle when horizontal wind was present. A similar decrease in climb angle in faster horizontal winds was previously reported for the miniature wasp *E. mundus* (Sarig and Ribak 2021) tested in the same horizontal wind tunnel. If ascending flight requires more power than horizontal flight, we may expect insects to reduce their climb angle when attempting to make progress upwind. Alternatively, when there is no wind miniature insects may prefer to fly vertically to reach horizontal air currents high above ground. In both scenarios, the climb angle is expected to decrease in the presence of wind. However, the facts that the insects showed preference to fly in the upwind direction and were reluctant to take-off at higher wind speeds better fit the first explanation.

The flights recorded in the horizontal wind tunnel were short because the insects quickly left the small area overlapped by the two high-speed cameras. As such they represent flights immediately after take-off. Prior to take-off, a perching insect can measure wind direction from the airflow relative to its stationary body. Once flying, however, it is less obvious if and how the insects can sense the direction of wind relative to their flight direction (Reynolds et al. 2016). One way to estimate wind direction during flight is to compare the ground velocity to aerial velocity. The aerial velocity can be measured by sensory bristles (Weis-Fogh 1949) or the antenna (Gewecke 1970; Gewecke and Heinzel 1980), while ground speed is typically estimated from vision using optic flow (Olberg 1981; Srinivasan et al. 2000; Baird et al. 2005; Lawson and Srinivasan 2018; Goyal et al. 2023). Miniature insects perceive the visual environment around them with reduced compound eyes (thrips: Ben-Yakir et al. 2023; E. mundus: Sarig and Ribak 2021; whiteflies: Urca and Lehmann 2025). Visual acuity is expected to diminish with smaller eyes and fewer ommatidia making vision a less reliable sense in very small insects (Land 1997; Makarova et al. 2019; Sarig and Ribak 2021). It should also become less effective at higher altitudes above the ground (Srinivasan et al. 2000; Reynolds et al. 2016). Therefore, it is possible that upwind flight direction was predetermined before take-off and once airborne the ability of the insects to sense the wind direction was eliminated. To test if the upwind flight was limited to take-off, we repeated the horizontal wind tunnel experiment while filming with the wider lens (28 mm). The recorded flights were indeed longer (duration) and included flight direction changes after take-off. They showed that although the insects did not make significant progress upwind relative to the ground, they were positioned well upwind relative to their expected position if they were merely drifting downwind (Supporting Information, SI4). Moreover, the data indicate a reluctance to actively fly in the downwind direction. Only four insects (one whitefly, one beetle, and two thrips) out of 34 moved downwind faster than predicted by passive drifting. Nevertheless, even in this larger tracking volume we were unable to track the insects in the horizontal wind tunnel over durations larger than 3 s. Therefore, we cannot determine if flight in the upwind direction persisted after the insects left the field of view.

Our wind tunnel set-up included a UV lamp positioned upstream. The lamp was intended to motivate the insects to fly upwind and thus reach faster flight speeds that better define their performance limit. In theory, the position of the lamp can explain the general preference of the insects to fly in the upwind direction. In practice, the same lamp was present and working in both the stagnant air and wind conditions of the experiments in both wind tunnels. Therefore, the change in flight direction observed here when wind was present resulted from the wind, not the lamp. Trials in the same horizontal wind tunnel with a lamp located crosswind showed that whiteflies flew toward the light with an upwind flight component (Supporting Information, SI5). Therefore, compensation for wind drift occurred in this species even when the lamp was not aligned with the upwind direction.

Wind velocity in the natural environments may frequently exceed the flight speed of all the insects studied here. However, measurements from a crop field where whiteflies are common, found that wind speeds 0.5 m above ground and within the vegetation can be less than 35% the wind speed above the plants’ canopy (Sarig and Ribak 2021). Thus, with the top horizontal flight speed observed here, the insect should be capable of moving upwind within the vegetation even in wind speeds 1–2 m/s. The whiteflies studied here and *E. mundus* studied previously (Sarig and Ribak 2021) increased flight speed to fly in the upwind direction. Both displayed above average top speed for their size (Fig. 6A). The beetles and thrips displayed average and below average top speed for their size, respectively (Fig. 6A), and did not increase their flight speed in response to wind. Hence, the behavioral response and its effect on drifting downwind seems to differ between species in accord with their flight performance. Similarly, flight performance affected the probability of taking off in higher wind speeds. The wind speeds tested here were below the maximum flight speeds of the insects because all the tested insects became increasingly reluctant to take-off as wind speed increased. The highest wind speed that permitted frequent take-offs was 47%, 49%, and 36% of whiteflies, thrips, and beetles, respectively (Table 1, Fig. 3). The wasp (*E. mundus*) continued to take-off (although at much lower numbers) at wind speed close to its maximal flight speed (Sarig and Ribak 2021), leading to the frequent downward drift visible in Fig. 2B. A reluctance of small insects to take-off in winds has been reported before in *Diachasmimorpha longicaudata* (Hymenoptera; Messing et al. 1997), *Frankliniella occidentalis* (Thripidae; Ben-Yakir and Chen 2008), and *Eretmocerus hayati* (Hymenoptera; Strickland et al. 2017). Thrips have also been reported to prefer flight during hours when wind is minimal (Ben-Yakir and Chen 2008). Presumably, such preference alleviates the need to consider the wind direction when controlling flight direction and ground speed.

Altogether, we found that flight speed in miniature insects is higher than expected for their small size and that their turning capabilities exceed those of larger animals. While the three insects studied here are unlikely to overcome wind speeds > 0.68 m/s they avoided the problem by not taking off at wind speeds exceeding 50% of their maximal flight speed. At lower wind speeds, they tended to fly in the upwind direction rather than drifting or actively flying downwind. Combined, these findings challenge the idea that miniature insects passively drift in winds and provide data on their flight performance that can be used to include active flight when predicting their dispersal.

## Supplementary Material

icag099_Supplemental_Files

## Data Availability

All data supporting the findings of this study are available within the paper and its supplementary information

## References

[bib1] Baird E, Srinivasan MV, Zhang S, Cowling A. 2005. Visual control of flight speed in honeybees. J Exp Biol. 208:3895–905. 10.1242/jeb.01818. 16215217

[bib2] Beatus T, Guckenheimer JM, Cohen I. 2015. Controlling roll perturbations in fruit flies. J R Soc Interface. 12:20150075. 10.1098/rsif.2015.0075. 25762650 PMC4387536

[bib3] Bejan A, Marden JH. 2006. Unifying constructal theory for scale effects in running, swimming and flying. J Exp Biol. 209:238–48. 10.1242/jeb.01974. 16391346

[bib4] Ben-Yakir D, Carvalho CJ. 2021. Using wind vanes to study how thrips reach colored traps. Entomol Exp Applicata. 169:1061–5. 10.1111/eea.13104.

[bib5] Ben-Yakir D, Chen M. 2008. Studies of thrips migratory flights in Israel. Acta Phytopathol Entomologica Hungarica. 43:243–8. 10.1556/APhyt.43.2008.2.5.

[bib6] Ben-Yakir D, van Tol RWHM, Bovio M, Ribak G. 2023. Distribution of Western flower thrips trapped on a yellow cylinder. J Insect Behav. 36:259–66. 10.1007/s10905-023-09838-3.

[bib7] Blackmer JL, Byrne DN. 1993a. Environmental and physiological factors influencing phototactic flight of Bemesia tabaci. Physiol Entomol. 18:336–42. 10.1111/j.1365-3032.1993.tb00606.x.

[bib8] Blackmer JL, Byrne DN. 1993b. Flight behaviour of *Bemisia tabaci* in a vertical flight chamber: effect of time of day, sex, age and host quality. Physiol Entomol. 18:223–32. 10.1111/j.1365-3032.1993.tb00592.x.

[bib9] Blackmer JL, Phelan PL. 1991. Behavior of *Carpophilus hemipterus* in a vertical flight chamber: transition from phototactic to vegetative orientation. Entomol Exp Applicata. 58:137–48. 10.1111/j.1570-7458.1991.tb01461.x.

[bib10] Bomphrey RJ, Walker SM, Taylor GK. 2009. The typical flight performance of blowflies: measuring the normal performance envelope of *Calliphora vicina* using a novel corner-cube arena. PLoS One. 4:e7852. 10.1371/journal.pone.0007852. 19924228 PMC2773008

[bib11] Bramwell ARS, Done G, Balmford D. 2001. Bramwell’s Helicopter Dynamics. 2nd ed Oxford: Butterworth Heinemann.

[bib12] Byrne DN . 1999. Migration and dispersal by the sweet potato whitefly, *Bemisia tabaci*. Agricult Forest Meteorol. 97:309–16. 10.1016/S0168-1923(99)00074-X. 28307104

[bib13] Chapman JW, Klaassen RHG, Drake VA, Fossette S, Hays GC, Metcale JD, Reynolds AM, Reynolds DR, Alerstam T. 2011. Animal orientation strategies for movement in flows. Curr Biol. 21:R861–70. 10.1016/j.cub.2011.08.014. 22032194

[bib14] Cheng X, Sun M. 2018. Very small insects use novel wing flapping and drag principle to generate the weight-supporting vertical force. J Fluid Mech. 855:646–70. 10.1017/jfm.2018.668.

[bib15] Cheng X, Sun M. 2021. Wing kinematics and aerodynamic forces in miniature insect *Encarsia formosa* in forward flight. Phys Fluids. 33:021905. 10.1063/5.0039911.

[bib16] Compton S . 2001. Sailing with the wind: dispersal by small flying insects. In: Bullock J, Kenward R, Hails R, editors. Dispersal ecology. Malden, MA, USA: Blackwell Publishing, Malden,MA.

[bib17] Corbett A, Rosenheim JA. 1996. Quantifying movement of a minute parasitoid, *Anagrus epos* (Hymenoptera: mymaridae), using fluorescent dust marking and recapture. Biol Control. 6:35–44. 10.1006/bcon.1996.0005.

[bib18] Desouhant E, Driessen G, Lapchin L, Wielaard S, Bernstein C. 2003. Dispersal between host populations in field conditions: navigation rules in the parasitoid *Venturia canescens*. Ecol Entomol. 28:257–67. 10.1046/j.1365-2311.2003.00511.x.

[bib19] Dickinson MH, Muijres FT. 2016. The aerodynamics and control of free flight maneuvers in *Drosophila*. Phil Trans R Soc B. 371:20150388. 10.1098/rstb.2015.0388. 27528778 PMC4992712

[bib20] Dudley R . 2002. Mechanisms and implications of animal flight maneuverability. Integr Comp Biol. 42:135–40. 10.1093/icb/42.1.135. 21708702

[bib21] Ellington CP . 1974. Non-steady-state aerodynamics of the flight of *Encarsia formosa*. In: Wu TY, Brokaw CJ, Brennen C, editors. Swimming and flying in nature. Vol 2:New York: Springer Science+Business Media. p.783–96.

[bib22] Farisenkov S, Kolomenskiy D, Petrov PN, Engels T, Lapina NA, Lehmann FO, Onishi R, Liu H, Polilov AA. 2022. Novel flight style and light wings boost flight performance of tiny beetles. Nature. 602:96–100. 10.1038/s41586-021-04303-7. 35046578 PMC8810381

[bib23] Farisenkov S, Lapina NA, Petrov PN, Polilov AA. 2020. Extraordinary flight performance of the smallest beetles. Proc Natl Acad Sci USA. 117:24643–5. 10.1073/pnas.2012404117. 32958659 PMC7547253

[bib24] Fernández-Moreno MA, Farr CL, Kaguni LS, Garesse R. 2007. *Drosophila melanogaster* as a model system to study mitochondrial biology. Methods Mol Biol. 372:33–49. 18314716 10.1007/978-1-59745-365-3_3 PMC4876951

[bib25] Fry SN, Sayaman R, Dickinson MH. 2003. The aerodynamics of free-flight maneuvers in *Drosophila*. Science. 300:495–8. 10.1126/science.1081944. 12702878

[bib26] Gewecke M . 1970. Antennae: another wind-sensitive receptor in Locusts. Nature 1970 225:5239 225:1263–4. 10.1038/2251263a0. 5435363

[bib27] Gewecke M, Heinzel H-G. 1980. Aerodynamic and mechanical properties of the antennae as air-current sense organs in *Locusta migratoria* I. Static characteristics. J Comp Physiol. 139:357–66. 10.1007/BF00610466.

[bib28] Goyal P, Baird E, Srinivasan MV, Muijres FT. 2023. Visual guidance of honeybees approaching a vertical landing surface. J Exp Biol. 226:jeb245956. 10.1242/jeb.245956. 37589414 PMC10482386

[bib29] Hardy AC, Milne PS. 1938. Aerial drift of insects. Nature 1938 141:602–3. 10.1038/141602a0.

[bib30] Hedrick TL . 2008. Software techniques for two- and three-dimensional kinematic measurements of biological and biomimetic systems. Bioinspir Biomim. 3:034001. 10.1088/1748-3182/3/3/034001. 18591738

[bib31] Hoerner SF . 1965. Fluid-dynamic drag.

[bib32] Horridge GA . 1956. The flight of very small insects. Nature. 178:1334–5. 10.1038/1781334a0.

[bib33] Isaacs R, Willis MA, Byrne DN. 1999. Modulation of whitefly take-off and flight orientation by wind speed and visual cues. Physiol Entomol. 24:311–8. 10.1046/j.1365-3032.1999.00144.x.

[bib34] Jones SK, Laurenza R, Hedrick TL, Griffith BE, Miller LA. 2015. Lift vs. drag based mechanisms for vertical force production in the smallest flying insects. J Theor Biol. 384:105–20. 10.1016/j.jtbi.2015.07.035. 26300066

[bib35] Kristensen NP, Schellhorn NA, Hulthen AD, Howie LJ, Barro PJDE. 2013. Wind-borne dispersal of a parasitoid: the process, the model, and its validation. Env Entom. 42:1137–48. 10.1603/EN12243. 24216288

[bib36] Land MF . 1997. Visual acuity in insects. Annu Rev Entomol. 42:147–77. 10.1146/annurev.ento.42.1.147. 15012311

[bib37] Lawson KKK, Srinivasan MV. 2018. Flight control of fruit flies: dynamic response to optic flow and headwind. J Exp Biol. 221:jeb189720. 10.1242/jeb.189720. 28314748

[bib38] Liu Y, Sun M. 2024. Aerodynamics and power requirements of climbing flight in fruit fly model. Phys Fluids. 36:41912. 10.1063/5.0198066.

[bib39] Lopez-Reyes K, Lankheet MJ, van Tol RWHM, Butler RC, Teulon DA, Armstrong KF. 2023. Tracking the flight and landing behaviour of western flower thrips in response to single and two-colour cues. Sci Rep. 13:14178. 10.1038/s41598-023-37400-w. 37648681 PMC10469208

[bib40] Lyu YZ, Sun M. 2022. Dynamic stability in hovering flight of insects with different sizes. Phys Rev E. 105:054403. 10.1103/PhysRevE.105.054403. 35706178

[bib41] Makarova AA, Meyer-rochow VB, Polilov AA. 2019. Morphology and scaling of compound eyes in the smallest beetles (Coleoptera : ptiliidae). Arthropod Struct Dev. 48:83–97. 10.1016/j.asd.2019.01.001. 30625373

[bib42] Maxworthy T . 1979. Experiments on the Weis-Fogh mechanism of lift generation by insects in hovering flight. Part 1. Dynamics of the “fling.”. J Fluid Mech. 93:47–63. 10.1017/S0022112079001774.

[bib43] McMasters JH, Henderson ML. 1980. Low-speed single-element airfoil synthesis. Tech Soaring. 6:1–21.

[bib44] Meng X, Liu Y, Sun M. 2017. Aerodynamics of ascending flight in fruit flies. J Bionic Eng. 14:75–87. 10.1016/S1672-6529(16)60379-7.

[bib45] Messing RH, Klungness LM, Jang EB. 1997. Effects of wind on movement of *Diachasmimorpha longicaudata*, a parasitoid of tephritid fruit flies, in a laboratory flight tunnel. Entomol Exp Applicata. 82:147–52. 10.1046/j.1570-7458.1997.00124.x.

[bib46] Olberg RM . 1981. Parallel encoding of direction of wind, head, abdomen, and visual pattern movement by single interneurons in the Dragonfly. J Comp Physiol. 142:27–41. 10.1007/BF00605473.

[bib47] Pasek JE . 1988. Influence of wind and windbreaks on local dispersal of insects. Agricult Ecosyst Environ. 22-23:539–54. 10.1016/0167-8809(88)90044-8.

[bib48] Polilov AA . 2015. Small Is beautiful: features of the smallest insects and limits to miniaturization. Annu Rev Entomol. 60:103–21. 10.1146/annurev-ento-010814-020924. 25341106

[bib49] Rayner JMV, Aldridge HDJN. 1985. Three-dimensional reconstruction of animal flight paths and the turning flight of microchiropteran bats. J Exp Biol. 118:247–65. 10.1242/jeb.118.1.247.

[bib50] Reynolds AM, Reynolds DR, Sane SP, Hu G, Chapman JW. 2016. Orientation in high-flying migrant insects in relation to flows: mechanisms and strategies. Phil Trans R Soc B. 371:20150392. 10.1098/rstb.2015.0392. 27528782 PMC4992716

[bib51] Ribak G, Dafni E, Gerling D. 2016. Whiteflies stabilize their take-off with closed wings. J Exp Biol. 219:1639–48. 27045098 10.1242/jeb.127886

[bib52] Roy CL, Debat V, Llaurens V. 2019. Adaptive evolution of butterfly wing shape: from morphology to behaviour. Biol Rev. 94:1261–81. 10.1111/brv.12500. 30793489

[bib53] Santhanakrishnan A, Robinson AK, Jones S, Low AA, Gadi S, Hedrick TL, Miller LA. 2014. Clap and fling mechanism with interacting porous wings in tiny insect flight. J Exp Biol. 217:3898–909. 10.1242/jeb.084897. 25189374

[bib54] Sarig A, Engels T, Lehmann F-O, Ribak G. 2025. Muscle power output reflects elevated viscosity in the propulsion system of flying miniature wasps. J R Soc Interface. 22:20250416. 10.1098/rsif.2025.0416. 41537870

[bib55] Sarig A, Ribak G. 2021. To what extent can the tiny parasitoid wasps, *Eretmocerus mundus*, fly upwind?. J Appl Entomol. 145:660–74. 10.1111/jen.12890.

[bib56] Simmons GS . 2000. Studies on dispersal of a native parasitoid Eretmocerus eremicus and augmentative biological control of Bemisia tabaci infesting cotton. Ph.D thesis, University of Arizona, Tucson.

[bib57] Srinivasan MV, Zhang SW, Chahl JS, Barth E, Venkatesh S. 2000. How honeybees make grazing landings on flat surfaces. Biol Cybern. 83:171–83. 10.1007/s004220000162. 11007294

[bib58] Strickland C, Kristensen NP, Miller L. 2017. Inferring stratified parasitoid dispersal mechanisms and parameters from coarse data using mathematical and Bayesian methods. J R Soc Interface. 14:20170005. 10.1098/rsif.2017.0005. 28539481 PMC5454288

[bib59] Sun M . 2023. Colloquium: miniature insect flight. Rev Mod Phys. 95:041001. 10.1103/RevModPhys.95.041001.

[bib60] Sun X, Gong X, Huang D. 2017. A review on studies of the aerodynamics of different types of maneuvers in dragonflies. Arch Appl Mech. 87:521–54. 10.1007/s00419-016-1208-7.

[bib61] Taylor L . 1974. Insect Migration, Flight Periodicity and the Boundary Layer. J Anim Ecol. 43:225–38. 10.2307/3169.

[bib62] Tennekes H . 2009. The Simple Science of Flight. Cambridge: MIT Press.

[bib63] Theriault DH, Fuller NW, Jackson BE, Bluhm E, Evangelista D, Wu Z, Betke M, Hedrick TL. 2014. A protocol and calibration method for accurate multi-camera field Videography. J Exp Biol. 217:1843–8. 24577444 10.1242/jeb.100529

[bib64] Urca T, Lehmann F-O. 2025. Visual gaze bias motion detection by split eyes in miniature whiteflies. iScience. 28:112730. 10.1016/j.isci.2025.112730. 40538434 PMC12177176

[bib65] Vosteen I, van der Meiracker N, Poelman E. 2020. Gone with the wind: low availability of volatile information limits foraging efficiency in downwind-flying parasitoids. Anim Behav. 165:59–70. 10.1016/j.anbehav.2020.04.025.

[bib66] Wainwright CE, Reynolds DR, Reynolds AM. 2020. Linking small-scale flight manoeuvers and density profiles to the vertical movement of insects in the nocturnal stable boundary layer. Sci Rep. 10:57779. 10.1038/s41598-020-57779-0. PMC6978332 31974508

[bib67] Wainwright CE, Stepanian PM, Reynolds DR, Reynolds AM. 2017. The movement of small insects in the convective boundary layer: linking patterns to processes. Sci Rep. 7: 5438. 10.1038/s41598-017-04503-0. 28710446 PMC5511248

[bib68] Weis-Fogh T . 1949. An aerodynamic sense organ stimulating and regulating flight in locusts. Nature. 164:873–4. 10.1038/164873a0. 15393878

[bib69] Weis-Fogh T . 1973. Quick estimates of flight fitness in hovering animals including novel mechanisms for lift production. J Exp Biol. 59:169–230. 10.1242/jeb.59.1.169.

[bib70] Weis-Fogh T . 1975. Unusual mechanisms for the generation of lift in flying animals. Sci Am. 233:80–7. 10.1038/scientificamerican1175-80. 1188343

