# Supplementary material for: Flight Performance and Flight in Wind in Three Phylogenetically Distinct Miniature Insects
Source: Integr Comp Biol. 2026 Jun 22;66:icag099. doi: 10.1093/icb/icag099 (PMC13393910; doi:10.1093/icb/icag099)
Supplement: icag099_Supplemental_Files [file icag099_supplemental_files.zip › icb-2026-0020-File010.docx]

**Supporting Information**

**Flight performance and flight in wind in three phylogenetically distinct miniature insects**

Amir Sarig, Lior Gurka and Gal Ribak

School of Zoology, Tel Aviv University, Israel

**Content:**

- Movie caption 01 – Flight in horizontal wind and the definition of upwind direction and displacement relative to the ground. The movie shows examples of a whitefly a thrips and a beetle flying upwind (towards the right) in the horizontal wind tunnel. The wind direction is from right to left and although the insects are flying in the upwind direction their displacement relative to the ground can be either upwind or downwind at specific flight segments depending on their flight speed and direction.
- Movie caption 02 – An example of the flight trajectory of a bark beetle flying upwards against a downward air flow of 0.1 m/s. The video shows the synchronized views from two cameras and the tracked trajectory is overlayed on the left camera view.
- Movie caption 03 – Manoeuvrability and flight in stagnant air. A slowed down movie showing a free flying whitefly from two camera views. The insect is flying in the working section of the horizontal wind tunnel while the wind is turned off (wind speed = 0). The flight trajectory includes multiple manoeuvres interspaced with steady flight and hovering.
- SI1: Maximal flight speeds of insects spanning two orders of magnitude in body size.
- SI 2 Extracting manoeuvres from flight trajectories
- SI 3 Manoeuvrability data from other animals
- SI 4 Effect of tracking duration and tracked volume on downwind drift in the horizontal wind tunnel
- SI 5 *Bemisia tabaci* compensate for wind drift during cross-wind flight
- Cited SI references

**SI1: Reported maximal flight speeds of insects varying in size.**

Table S1. The data used to generate Figure 6A of the main text and its sources. The reference list can be found at the end of this SI file. The three right columns provide information on methodology including obstruction due to arena size (in body lengths), the measurement technique and the temporal resolution of measurement when speed was derived from position data. Only studies citing maximal flight speed or providing a range of flight speeds enabling to estimate top speed are mentioned.

| **Order**  Species | | **BL (mm)** | | | **Max** $\boldsymbol{V}_{\boldsymbol{xy}}$ | **Re** | | $\boldsymbol{V}_{\boldsymbol{xy}}$ **source** | | | **Enclosure volume (BL)^3^** | **Method** | | | **Measurement frequency (Hz)** |  | |  |  |  |
| --- | --- | --- | --- | --- | --- | --- | --- | --- | --- | --- | --- | --- | --- | --- | --- | --- | --- | --- | --- | --- |
| **Coleoptera** | |  | | |  |  | |  | | |  |  | | |  |  | |  |  |  |
| *Nanosella sp.* | | 0.37 | | | 0.16 | 4 | | Farisenkov et al. 2020 | | | >70^3^ | Video | | | 200 |  | |  |  |  |
| *Paratuposa placentis* | | 0.38 | | | 0.21 | 5 | | Farisenkov et al. 2020 | | | >70^3^ | Video | | | 240 |  | |  |  |  |
| *Limulosella waspucensis* | | 0.4 | | | 0.19 | 5 | | Farisenkov et al. 2020 | | | >70^3^ | Video | | | 240 |  | |  |  |  |
| *Mikado sp.* | | 0.46 | | | 0.26 | 8 | | Farisenkov et al. 2020 | | | >70^3^ | Video | | | 200 |  | |  |  |  |
| *Nephanes titan* | | 0.6 | | | 0.52 | 20 | | Farisenkov et al. 2020 | | | >70^3^ | Video | | | 240 |  | |  |  |  |
| *Acrotrichis sericans* | | 0.9 | | | 0.88 | 53 | | Farisenkov et al. 2020 | | | >70^3^ | Video | | | 300 |  | |  |  |  |
| *Ptenidium pusillum* | | 1 | | | 0.49 | 33 | | Farisenkov et al. 2020 | | | >70^3^ | Video | | | 240 |  | |  |  |  |
| *Acrotrichis grandicolli* | | 1.03 | | | 0.66 | 45 | | Farisenkov et al. 2020 | | | >70^3^ | Video | | | 240 |  | |  |  |  |
| *Hypoborus ficus* | | 1.3 | | | 0.60 | 50 | | This study | | | Wind tunnel | Video | | | 60 |  | |  |  |  |
| *Gyrophaena sp.1* | | 1.43 | | | 0.94 | 90 | | Farisenkov et al. 2020 | | | >70^3^ | Video | | | 150 |  | |  |  |  |
| *Atheta sp.* | | 2.06 | | | 0.5 | 69 | | Farisenkov et al. 2020 | | | >70^3^ | Video | | | 300 |  | |  |  |  |
| *Euwallacea nr. Fornicatus*^A^ | | 2.7 | | | 0.6 | 108 | | Calnaido 1965 | | | Empty room | distance/time | | | NA |  | |  |  |  |
| *Gyrophaena sp. 2* | | 2.92 | | | 0.63 | 123 | | Farisenkov et al. 2020 | | | >70^3^ | Video | | | 100 |  | |  |  |  |
| *Dinaraea sp.* | | 3.11 | | | 0.71 | 147 | | Farisenkov et al. 2020 | | | >70^3^ | Video | | | 300 |  | |  |  |  |
| *Lordithon lunulatus* | | 6.16 | | | 1.91 | 784 | | Farisenkov et al. 2020 | | | >70^3^ | Video | | | 100 |  | |  |  |  |
| *Philonthus sp.* | | 11.97 | | | 1.36 | 1085 | | Farisenkov et al. 2020 | | | >70^3^ | Video | | | 100 |  | |  |  |  |
| *Oiceoptoma thoracicum* | | 15.39 | | | 1.89 | 1940 | | Farisenkov et al. 2020 | | | >70^3^ | Video | | | 100 |  | |  |  |  |
| *Nicrophorus vespillo* | | 21.04 | | | 1.66 | 2328 | | Farisenkov et al. 2020 | | | >70^3^ | Video | | | 100 |  | |  |  |  |
| *Nicrophorus investigator* | | 21.07 | | | 2.33 | 3273 | | Farisenkov et al. 2020 | | | >70^3^ | Video | | | 100 |  | |  |  |  |
| *Protaetia cuprea*^&^ | | 23 | | | 2.28 | 3496 | | Urca et al. 2022 | | | 87×65×109 | Video | | | 125 |  | |  |  |  |
| *Trypoxylus dichotomus*^B^ | | 40 | | | 4.0 | 10666 | | McCullough & Tobalske 2013 | | | Outdoor | Radar gun | | | NA |  | |  |  |  |
| **Diptera** | |  | | |  |  | |  | | |  |  | | |  |  | |  |  |  |
| *Drosophila melanogaster^C^* | | | 3 | 0.85 | | | 170 | | Marden et al. 1997 | | 166^3^ | | Video | | 60 |  | |  |  |  |
| *Drosophila melanogaster*^C^ | | | 3 | 1.6 | | | 320 | | Ray et al. 2016 | | 666×666×600 | | Video | | 500 |  | |  |  |  |
| *Drosophila virillis*^D^ | | | 2.25 | 2 | | | 300 | | Vogel 1996 cf. Dudley 2000 | | Wind tunnel | | Wind speed | | NA |  | |  |  |  |
| *Cyrtodiopsis dalmanni* | | | 6.5 | 0.3 | | | 130 | | Ribak & Swallow 2007 | | 154×107×107 | | Video | | 60 |  | |  |  |  |
| *Calliphora vicina^E^* | | | 8.83 | 1.2 | | | 706 | | Schilstra & Van Hateren 1999 | | 45×45×45 | | Search coil | | 1000 |  | |  |  |  |
| *Calliphora vicina^E^* | | | 8.83 | 2.4 | | | 1413 | | Bomphrey et al. 2009 | | 181^3^ | | Video | | 250/500 |  | |  |  |  |
| **Hemiptera** | | |  |  | | |  | |  | |  | |  | |  |  | |  |  |  |
| *Bemisia tabaci* | | | 0.81 | 0.68 | | | 37 | | This study | | Wind tunnel | | video | | 60 |  | |  |  |  |
| **Hymenoptera** | | |  |  | | |  | |  | |  | |  | |  |  | |  |  |  |
| *Eretmocerus eremicus*^F^ | | | 0.6 | 0.16 | | | 6 | | Byrne 1999 | | Wind tunnel | | Wind speed | | NA |  | |  |  |  |
| *Eretmocerus mundus^G^* | | | 0.7 | 0.5 | | | 23 | | Sarig & Ribak 2021 | | Wind tunnel | | Video | | 5000 |  | |  |  |  |
| *Bombus terrestris* (worker)^H^ | | | 14 | 9.7^&^ | | | 9000 | | Riley et al. 1999 | | Outdoor | | Radar | | NA |  | |  |  |  |
| *Bombus pascuorum^H^* | | | 12 | 5 | | | 4000 | | Ellington et al. 1990 | | Wind tunnel | | Wind speed | | NA |  | |  |  |  |
| **Lepidoptera** | | |  |  | | |  | |  | |  | |  | |  |  | |  |  |  |
| *Urania fulgens* | | | 22.1 | 4.2 | | | 6216 | | Dudley & DeVries 1990 | | Outdoor | | Video | | 30 |  | |  |  |  |
| **Odonata** | | |  |  | | |  | |  | |  | |  | |  |  | |  |  |  |
| *Sympetrum danae*^I^ | | | 32 | 4 | | | 8533 | | Rüppell 1989 | | Outdoor | | Video | | unspecified |  | |  |  |  |
| *Sympetrum sanguineum*^I^ | | | 34 | 1.66 | | | 3762 | | Wakeling & Ellington 1997 | | Large greenhouse | | Video | | 3000 |  | |  |  |  |
| *Chalcolestes viridis^J^* | | | 44.8 | 2.3 | | | 6869 | | Rüppell 1989 | |  | | Video | | unspecified |  | |  |  |  |
| *Calopteryx splendens*^I^ | | | 45 | 1.9 | | | 5700 | |  | |  | |  | |  |  | |  |  |  |
| *Anas junius*^D^ | | | 66 | 7.5 | | | 33000 | | Ruppel 1989 cf Dudley 2000 | | Outdoor | | Video | | unspecified |  | |  |  |  |
| **Orthoptera** | | |  |  | | |  | |  | |  | |  | |  |  | |  |  |  |
| *Locusta migratoria*^D^ | | | 40 | 4.6 | | | 12267 | | Baker et al. 1981 cf Dudley 200 | | Outdoor | | Video | | 500 |  | |  |  |  |
| **Thysanoptera** | | |  |  | | |  | |  | |  | |  | |  |  | |  |  |  |
| *Frankliniella occidentalis*^K^ | | | 1.59 | 0.27 | | | 29 | | Ben-Yakir et al. 2023 | | Wind tunnel | | Wind speed | | NA |  | |  |  |  |
| *Gynaikothrips ficorum* | | | 2.2 | 0.46 | | | 67 | | This study | | Wind tunnel | | Video | | 60 |  | |  |  |  |
| ^A^ Body length from Smith et al. 2019 | | | | | | |  | | ^E^ Body length from Salanitro et al. 2022 | | |  | | | | |  |  |  |  |
| ^B^ Body length from Mccullough et al. 2012 | | | | | | |  | | ^F^ Body length from Rose & Zolnerowich 1997 | | |  |  |  |  |  |  |  |  |  |
| ^C^ Body length from Fernández-Moreno et al. 2007 | | | | | | |  | | ^G^ Body length from Mercet 1931 | |  |  | |  | | |  |  |  |  |
| ^D^ Body length calculated from speed and Re number | | | | | | |  | | ^H^ Body length from Alford 1975 | |  |  | |  | | |  |  |  |  |
| ^I^ Body length from: <https://maps.biodiversityireland.ie/> (National Biodiversity Data Centre, Ireland) | | | | | | | | | | | | | | | | |  |  |  |  |
| ^J^ Body length from Gyulavári et al. 2011 | | | | | | |  | |  |  |  |  | |  | | |  |  |  |  |
| ^K^ Body length from De Kogel et al. 2013 | | | | | | |  | |  |  |  |  | |  | | |  |  |  |  |
| ^&^Maximal flight speed unavailable, mean + 2 SD used instead | | | | | | | | | |  |  |  | |  | | |  |  |  |  |
|  | | | | | | | | | | |  |  | |  | | |  |  | |  |
|  | | | | | | | | | |  |  |  | |  | | |  |  |  |  |
|  | | | | | | |  | |  |  |  |  | |  | | |  |  |  |  |
|  | | | | | | | | | |  |  |  | |  | | |  |  |  |  |
|  | | | | | | | | |  |  |  |  | |  | | |  |  |  |  |
|  |  | |  |  | | |  | |  |  |  |  | |  | | |  |  |  |  |

**SI 2 extracting manoeuvres from flight trajectories**

**
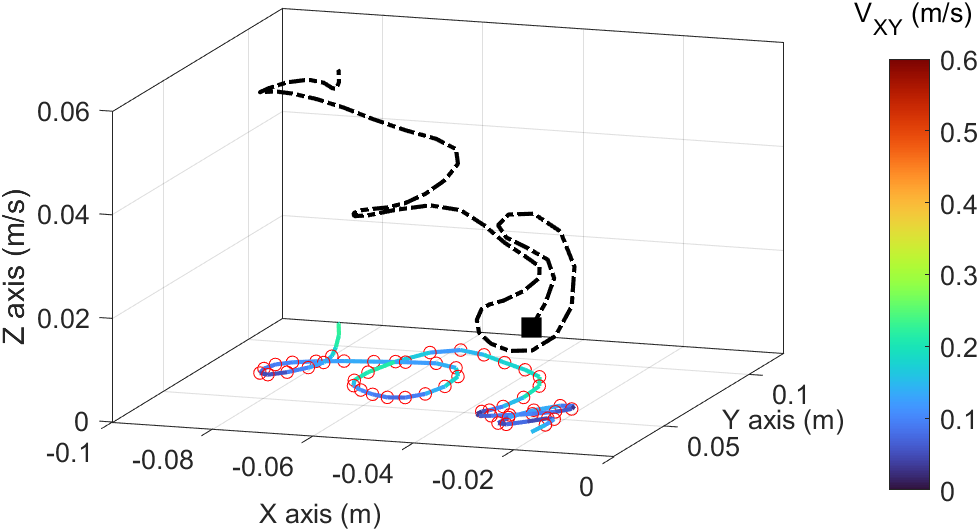
**

**Figure SI 2: 3D Extracting manoeuvres from flight trajectory.** Example of the 3D flight trajectory of a whitefly in the horizontal wind tunnel (dashed black line). The start of the trajectory is denoted by a black square. The horizontal (2D) projection of the trajectory is colour coded according to horizontal flight speed ($V_{XY}$). Red circles denote video frames identified as curves in the flight trajectory and used to identify manoeuvres for the manoeuvrability analysis.

**SI 3 Manoeuvrability data**

**Table SI 3.1 Data used to generate figure 6A and 6B and their sources.**

| **Species** | **Number of Manoeuvres** | **Body length (m)** | **Data source** |
| --- | --- | --- | --- |
| *Bemisia tabaci* | 324 | 0.0008 | This study |
| *Hypoborus ficus* | 674 | 0.0013 | This study |
| *Gynaikothrips ficorum* | 229 | 0.0022 | This study |
| *Dineutes horni* | 110 | 0.0124 | Fig. 4 in Fish & Nicastro 2003 |
| *Scarabaeus puncticollis^A^* | 32 | 0.0180 | Unpublished data, described below |
| *Copris hispanus^A^* | 45 | 0.0225 | Unpublished data, described below |
| *Protaetia cuprea^B^* | 32 | 0.023 | Unpublished data, described below |
| *Calypte anna^C^* | 4 | 0.100 | Fig. 1 in Read et al. 2016 |
| *Calypte anna^C^* | 52 | 0.100 | Segre et al., 2015 uploaded data |
| *Acinonyx jubatus^D^* | *198* | 0.708 | Fig 4e in Wilson et al. 2013 |
| *Phalcrocorax carbo* | *6* | 0.850 | Fig, 4 in Ribak et al. 2008 |
| *Zalophus californianus* | 87 | 1.805 | Fig. 1 in Fish et al. 2003 |

^A^ body length taken from Rittner (online source, see reference)

^B^ body length taken from Urca et al. 2022

*^C^* body length taken from Cornell Lab of Ornithology 2019

^D^ body length taken from Hudson et al. 2012

Unpublished turning performance for three Scarab beetles.

Fig 6A and 6B of the main text include unpublished data on the turning performance of three scarab beetles. The methodology used to collect that data was:

*Protaetia cuprea* and *Copris hispanus*, were collected from various field sites at Northern Israel, and *Scarabaeus puncticollis* were collected from Nitzanim nature reserve in Southern Israel. All insects were recorded at 1000 FPS using two synchronised high-speed cameras (Fastcam SA3, Photron Inc.) while flying in a large room ($4.5 \times4.5 \times3.5 m, W \times L \times H$) around a mercury vapor light-bulb located at the room's centre. The resulting flight trajectories were primarily circular flights around the lamp. We calculated the instantaneous flight speed and curvature in the horizontal plane for each beetle. Next, we binned the data to 60 Hz time intervals, as described in the main text. Then, we found the median values of the flight speed and turning radius for each manoeuvre. These medians are used in Figure 6B and 6C of the main text and available in the supporting data file. Table S3.2 denotes the species-specific sample sizes, medians and SD.

**Table SI 3.2: Number of beetles, number of manoeuvres and medians of turning performance for three Scarab beetles.** The data of the turning radius and flight speed are Median (±SD)**.**

| Species | N (beetles) | N (manoeuvres) | Turning radius (m) | Flight speed (m/s) |
| --- | --- | --- | --- | --- |
| *Protaetia cuprea* | 16 | 32 | 0.313 (0.253) | 0.795 (0.598) |
| *Copris hispanus* | 12 | 45 | 0.255 (0.416) | 0.86 (0.41) |
| *Scarabaeus puncticollis* | 19 | 32 | 0.247 (2.819) | 0.923 (0.463) |

**SI 4 Effect of tracking duration and tracked volume on downwind displacement (drift) in the horizontal wind tunnel**

**Fig S4.1 Effect of tracking volume on downwind displacement.** To distinguish between take-off direction and persistent upwind flight we compared the position of the insects in the horizontal wind tunnel at the end of the tracked flight trajectory (full circles) with their expected position for drifting passively downwind (open circles). Figures on the left corresponds to take-off behaviour tracked within a small field of view (filmed with a 85 mm lens). Figures on the right corresponds to tests in the same horizontal wind tunnel and same wind speeds but the insects were tracked within a larger field of view (28 mm lens). The wind speeds were 0.23, 0.12 and 0.14 m/s for the whiteflies, thrips and beetles, respectively. The upper panel shows the duration of tracked flight trajectory before the insect left the field of view of the two cameras. Lower panel shows the up/downwind final position of the insect in the wind tunnel relative to their take-off point (i.e., the displacement relative to the ground). The black dashed line divides the data to insects located up/downwind of the take-off point. Colour denotes species according to the legend at the top of the figure.

**
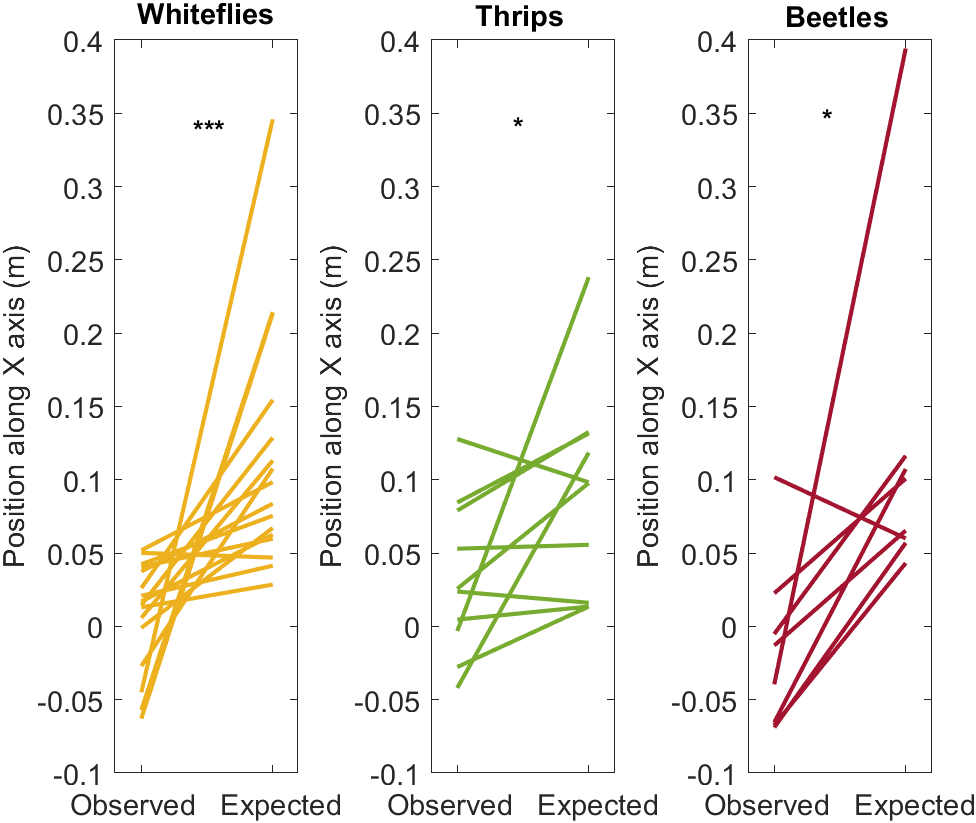
**

**Fig S4.2 Observed position vs. expected position for drifting downwind.** The data are the same as in Fig S4.1. Each line corresponds to a single insect. The vertical axis is the up/downwind displacement of the insect relative to the ground between the starting and at the end of tracking. Positive and negative values correspond to downwind and upwind displacements respectively. The expected position for each insect is the wind speed x track duration. Asterisks denote statistical significance in a Wilcoxon signed rank test (* p<0.05, *** p<0.001)

**SI 5 *Bemisia tabaci* compensates for wind drift during cross-wind flight**

The flight of *B. tabaci* in the horizontal wind tunnel towards a light source located cross wind was filmed (250 fps) with the two spatially calibrated high-speed cameras through 50 mm Nikon lenses. The insects were taken from the same population and transferred to the lab in the same manner described in the Method section of the main text. The pipette housing the insects protruded from the wall of the wind tunnel and a green + UV cylindrical light source (Length = 4 cm, diameter = 1 cm) was mounted horizontally 11 cm away, on the opposite wall of the test section (Fig. S5A). We tracked the flight trajectories of 58 insects from takeoff to landing or exiting the cameras' field of view. Twenty-one of the tracked trajectories were in 'no wind' conditions and the other 37 with the wind tunnel fan turned on (wind speed 0.12 m/s). The position of the insect at the end of the tracked trajectory was subtracted from the position at take-off and compared to the expected position if the insect only flew cross wind while drifting passively downwind.

**
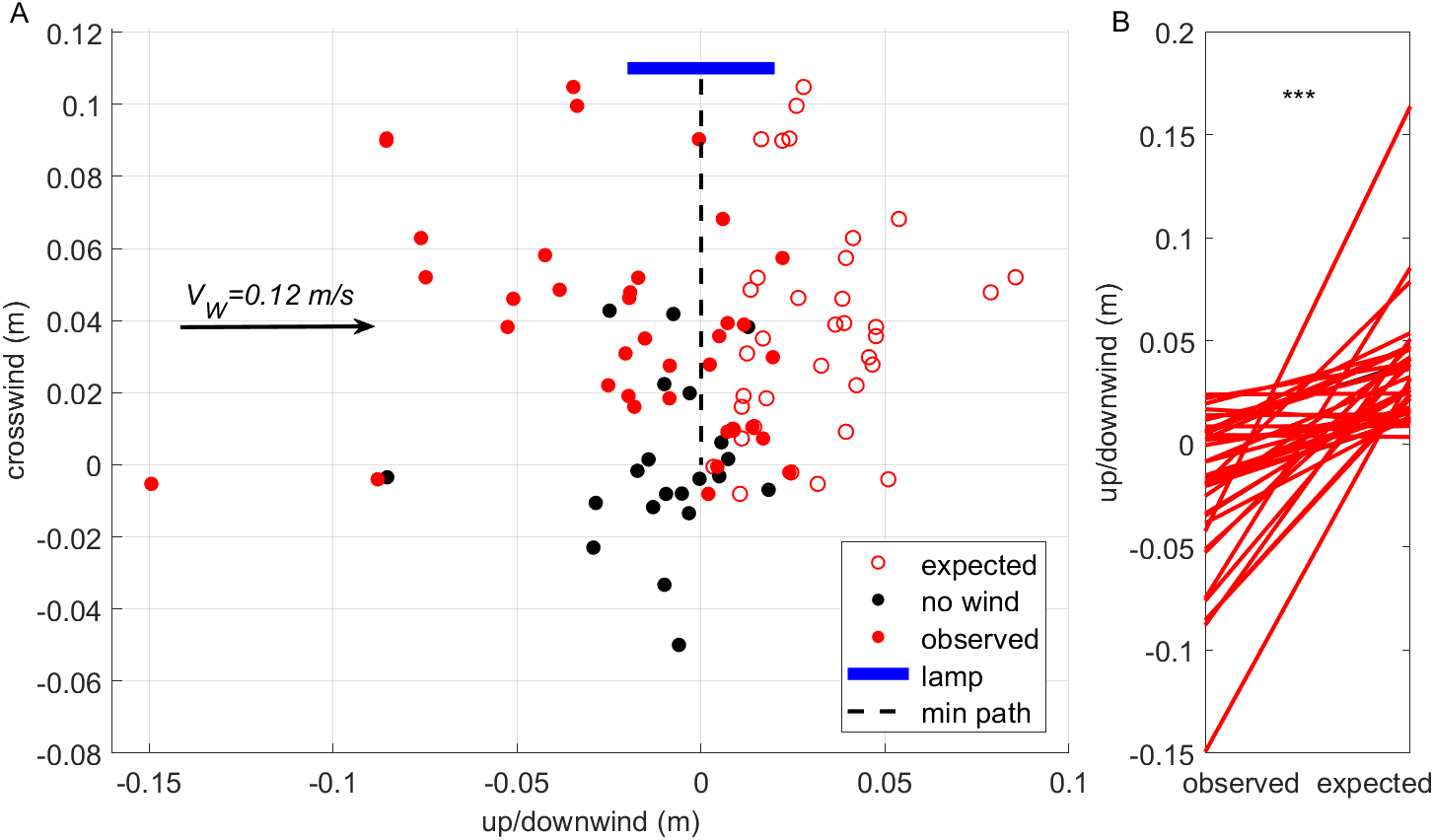
Fig S5 crosswind flight.** A) Each circle is the position of the whiteflies in the horizontal wind tunnel at the end of the tracked flight trajectory (full circles). The take-off position for all the insects is (0,0). Black and red circles denote trials with no wind (n=21) and 0.12 m/s wind (n=37), respectively. The location and size of the lamp is denoted by the thick blue line. Open red circles denote the expected position for insects flying in wind without compensating for wind drift. Dashed black line shows the shortest path to the lamp and divides the data into insects displaced up/downwind relative to the ground. B) Same data for the 0.12 m/s crosswind as in (A) showing observed versus expected displacements. Each line corresponds to a single insect (n=37). The vertical axis is the up/downwind displacement of the insect relative to the ground at the end of tracking. Positive and negative values correspond to downwind and upwind displacement respectively. The expected displacement for each insect is the wind speed x track duration. Asterisks denote statistical significance in a paired t-test (*** p<0.001)

**Cited SI References**

Alford DV. 1997. *Bumblebees*. Davis-Poynter. London.

Baker P, Gewecke M, Cooter R. 1981. The natural flight of the migratory locust, *Locusta migratoria* L. - III. Wing-beat frequency, flight speed and attitude. J Comp Physiol A 141:233-237.

Ben-Yakir D, van Tol RW, Bovio M, Ribak, G. 2023. Distribution of Western flower thrips trapped on a yellow cylinder. J Insect Behav 36:259-266.‏

Bomphrey R, Walker S, Taylor G. 2009. The typical flight performance of blowflies: measuring the normal performance envelope of *Calliphora vicina* using a novel corner-cube arena. PLoS One 4, e7852

Byrne, DN. 1999. Migration and dispersal by the sweet potato whitefly, *Bemisia tabaci*, Agricultural and Forest Meteorology 97:309-316.

Calnaido D. 1965. The flight and dispersal of shot‐hole borer of tea (*Xyleborus fornicatus* Eichh., Coleoptera: Scolytidae). Entomologia Experimentalis et Applicata, 8:249-262.

De Kog WJ, Bosco D, Van Der Hoek M, Mollema C.  2013. Effect of host plant on body size of *Frankliniella occidentalis* (Thysanoptera: Thripidae) and its correlation with reproductive capacity. EJE, 96(4), 365-368.‏

Dudley R. 2000. The Biomechanics of Insect Flight. *Princeton, NJ: Princeton University Press*.

Dudley R, DeVries PJ. 1990. Flight physiology of migrating *Urania fulgens* (Uraniidae) moths: kinematics and aerodynamics of natural free flight. J Comp Physiol A 167:145-154.‏

Ellington CP, Machin KE, Casey TM. 1990. Oxygen consumption of bumblebees in forward flight. Nature 347, 472-473.

Farisenkov SE, Lapina NA, Petrov PN, Polilov AA. 2020. Extraordinary flight performance of the smallest beetles. Proc Nat Acad Sci 117:24643-24645.‏

Fernández-Moreno MA, Farr CL, Kaguni LS, Garesse R. 2007. *Drosophila melanogaster* as a model system to study mitochondrial biology. Mitochondria: Practical Protocols, 33-49.‏

Fish FE, Hurley J, Costa DP. 2003. Maneuverability by the sea lion *Zalophus californianus*: turning performance of an unstable body design*.* J Exp Biol, 206:667-674.

Fish FE, Nicastro AJ. 2003. Aquatic turning performance by the whirligig beetle: constraints on maneuverability by a rigid biological system. J Exp Biol 206:1649-1656.

Gyulavári HA, Felföldi T, Benken T, Szabó LJ, Miskolczi M, Cserháti C, Horvai V, Márialigeti K, Dévai G. 2011. Morphometric and molecular studies on the populations of the damselflies *Chalcolestes viridis* and *C. parvidens* (Odonata, Lestidae). Int J Odonat 14:329-339.

Hudson PE, Corr SA, Wilson AM. 2012. High speed galloping in the cheetah (*Acinonyx jubatus*) and the racing greyhound (*Canis familiaris*): spatio-temporal and kinetic characteristics. J Exp Biol 215: 2425-2434.‏

Marden JH, Wolf MR, Weber KE. 1997. Aerial performance of *Drosophila melanogaster* from populations selected for upwind flight ability. J Exp Biol 200:2747-2755.‏

McCullough EL, Tobalske BW. 2013. Aerodynamic costs Elaborate horns in a giant rhinoceros beetle incur negligible aerodynamic costs. Proc R Soc B *280*:20130197.

Mccullough EL, Weingarden PR, Emlen DJ. 2012. Costs of elaborate weapons in a rhinoceros beetle: how difficult is it to fly with a big horn? Behav Ecol 23:1042-1048.

Mercet RG. 1931. Notas sobre Aphelinidos (Hym. Chalc.), 4a nota. *Eos, Revista Española di Entomología*, Madrid, 7, 395.

Ray RP, Nakata T, Henningsson P, Bomphrey RJ. 2016. Enhanced flight performance by genetic manipulation of wing shape in *Drosophila*. Nat Comm 7:10851.‏

Read TJ, Segre PS, Middleton KM, Altshuler DL. 2016. Humming birds control turning velocity using body orientation and turning radius using asymmetrical wingbeat kinematics. J R Soc, Interface 13:20160110.‏

Ribak G, Swallow JG. 2007. Free flight maneuvers of stalk-eyed flies: do eye-stalks affect aerial turning behavior? J Com Physiol A 193:1065-1079.‏

Ribak G, Weihs D, Arad Z. 2008. Consequences of buoyancy to the maneuvering capabilities of a foot-propelled aquatic predator, the great cormorant (*Phalcrocorax carbo sinensis*). J Exp Biol 211:3009-3019.‏

Riley JR, Reynolds DR, Smith AD, Edwards AS, Osborne JL, Williams IH, McCartney HA 1999. Compensation for wind drift by bumble-bees. *Nature,* 400:126.

Rose M, Zolnerowich G. 1997. *Eretmocerus Haldeman* (Hymenoptera: Aphelinidae) in the United States, with descriptions of new species attacking *Bemisia* (Tabaci complex) (Homoptera: Aleyrodidae). Proc Entomol Soc Washington, 99:1–27. Specimen USNM repository: <http://n2t.net/ark:/65665/3e2e8e8fb-d15d-4d2a-9d5c-7233f6f8356b>

Rüppell G. 1989. Kinematic analysis of symmetrical flight manoeuvres of Odonata. J Exp Biol 144:13-42.‏

Salanitro LB, Massaccesi AC, Urbisaglia S, Peria ME, Centeno ND, Chirino MG. 2022. *Calliphora vicina* (Diptera: Calliphoridae): Growth rates, body length differences, and implications for the minimum post-mortem interval estimation *Revista de la Sociedad Entomológica Argentina*, 81(2).‏

Sarig A, Ribak G. 2021. To what extent can the tiny parasitoid wasps, *Eretmocerus mundus*, fly upwind? J App Entomol 145:660-674.

Schilstra C, Van Hateren JH. 1999. Blowfly flight and optic flow: I. Thorax kinematics and flight dynamics. J Exp Biol 202:1481-1490.‏

Segre PS, Dakin R, Zordan VB, Dickinson MH, Straw AD, Altshuler DL. 2015. Burst muscle performance predicts the speed, acceleration, and turning performance of Anna’s hummingbirds. Elife 4 e11159.‏ [Dataset]. Dryad. <https://doi.org/10.5061/dryad.14762>

Smith SM, Gomez DF, Beaver RA, Hulcr J, Cognato AI. 2019. Reassessment of the species in the *Euwallacea fornicatus* (Coleoptera: Curculionidae: Scolytinae) complex after the rediscovery of the ‘lost’ type specimen. Insects 2019:261.

Urca T, Levin E, Ribak G. 2022. Metabolic cost of flight and aerobic efficiency in the rose chafer, *Protaetia cuprea* (Cetoniinae). Insect Sci 29, 1361-1372.‏

Wilson AM, Lowe JC, Roskilly K, Hudson PE, Golabek KA, McNutt JW. 2013. Locomotion dynamics of hunting in wild cheetahs. Nature 498:185-189.‏

Online resources

National Biodiversity Data Centre, Ireland. (n.d.). *Sympetrum danae*. <https://species.biodiversityireland.ie/profile.php?taxonId=78188&taxonName=Sympetrum%20danae>. Accessed on 12 February 2024.

Rittner, O. (n.d.). Scarabaeidae: Scarabaeinae. Israel Nature Site. <https://israel-nature-site.com/?page_id=210>. Accessed on 05 February 2024.

Cornell Lab of Ornithology. 2019. All About Birds - Anna's Hummingbird. Cornell Lab of Ornithology, Ithaca, New York. [https://www.allaboutbirds.org/guide/Annas_Hummingbird/id#](https://www.allaboutbirds.org/guide/Annas_Hummingbird/id) . Accessed on 30 January 2024.
